# Women, peace and insecurity: The risks of peacebuilding in everyday life for women in Sri Lanka and Nepal

**DOI:** 10.1371/journal.pone.0303023

**Published:** 2024-05-29

**Authors:** Karen Brounéus, Erika Forsberg, Prakash Bhattarai, Neloufer de Mel, Kate Lonergan, Pradeep Peiris, Pawan Roy, Gameela Samarasinghe, Maneesha Wanasinghe-Pasqual

**Affiliations:** 1 Department of Peace and Conflict Research, Uppsala University, Uppsala, Sweden; 2 Centre for Social Change (CSC), Kathmandu, Nepal; 3 University of Colombo, Colombo, Sri Lanka; Dhaka University, BANGLADESH

## Abstract

Truth telling processes, initiatives to prosecute war-time perpetrators, and ex-combatant reintegration are examples of common peacebuilding practices after war. Yet, little is known of how women are affected by peacebuilding initiatives such as these, or how they perceive these initiatives for peace. For many women, peace after war does not bring peace to everyday life; research shows that domestic violence increases during and after war. In addition, some peacebuilding measures have been found to increase risk and insecurity, not least for women. To better understand the interconnections between gender and post-conflict attitudes to peacebuilding, we asked 2,041 women and men in Sri Lanka and Nepal of their views on post-war peace initiatives. In line with our expectations, we find that women are more skeptical than men towards peacebuilding measures that involve increased risk in everyday life, such as truth-telling and coexisting with former adversaries and warring groups reintegrating in local communities. There are no gender differences pertaining to peacebuilding initiatives that take place far away at the national level, for example, concerning accountability or, in the case of Nepal, the peace agreement. Our findings suggest that international peacebuilding practice is blind to the everyday insecurities of women after war. That a basic gendered lens is missing from most peacebuilding designs is both alarming and deeply troubling, but identifying this critical aspect provides the opportunity for imperative change. By shedding light on the challenges women face after war, we hope this article contributes to finding ways to mitigate unknown and unintended side-effects of peacebuilding efforts, and thereby to the development of better, evidence-based peacebuilding practice–of benefit to both men and women.

## Introduction

War, and the legacy of war, is gendered [1–5]. This gendered pattern of war and peacebuilding relates to aspects such as who participates in warfare, who is victimized, and how. It relates to all phases of war: building up to war, regimes often increase military spending and deprioritize women’s welfare [6]. During war, many women (but also men) are the targets of conflict-related sexual violence [7]. After war, the long-lasting physical and psychological health problems of war affect women disproportionately, due to societal norms or by a war-shattered infrastructure that hits hard on, for example, maternal health [8–11].

However, less is known about women’s experiences of post-war initiatives for peace. Research shows that certain initiatives for peacebuilding create risk and insecurity for women. For example, in Rwanda, Brounéus [12] found that women survivors who witnessed in the *gacaca* courts–the nationwide, village-based truth and reconciliation process initiated by the Rwandan government after the 1994 genocide–were subjected to threats and harassment before, during, and after their testimony. Some women were killed for telling their stories. Further, survey interviews at the time showed that Rwandan women were significantly more negative than men towards the gacaca process, and towards coexistence with former enemies [8]. Also in the Solomon Islands, in many ways a contrasting case to Rwanda, women were significantly more negative than men towards, for example, reintegrating ex-combatants after the armed conflict known as the *Tensions* [9].

These findings, that women were more skeptical to certain kinds of peacebuilding initiatives than men, are unexpected because the reigning paradigm has been that women are more supportive of peace than men. The ‘women and peace’ hypothesis has indeed long been an important influence in both scholarly and popular thinking around gender, war, and peace [13]. However, this hypothesis has been based mainly on research conducted in Western countries in peace [13–16], not in developing countries that have lived through war. That women in such contrasting cases as Rwanda and Solomon Islands were skeptical towards key peacebuilding initiatives suggests that these processes involve challenges that affect women negatively–perhaps in other post-conflict settings, too.

The research project behind this article springs out of these findings in Rwanda and Solomon Islands. By assuming that women are more supportive of peace also in post-war settings full stop, we may fail to see the actual consequences peacebuilding initiatives have for women after war. A multitude of differences exist between Rwanda and Solomon Islands and the wars in each place, but one common denominator exists: in both places, rape and other forms of sexual violence against women were commonplace both during and after the war. This is and has been the case in the majority of wars, today and through history [17], although significant variation between different wars show that such violence is not unavoidable [18]. War-related sexual violence has devastating effects on women’s health and life long after the war has ended [11, 19]. As in other post-war settings, there was a higher prevalence of psychological ill-health among women than men in both Rwanda and in the Solomon Islands, still many years after the end of war [9]. To note here, recent research has importantly uncovered that sexual violence against men during war is more frequent than previously known, with devastating effects [20–22]. In this article, however, we focus on disentagling why women—on average—demonstrate higher levels of psychological ill-health and less support to certain types of peacebuilding measures than men after war.

Intimate partner violence (IPV) against women increases during and after war [23, 24]. So, in war and after, women face not only the threats and devastating effects of war for their families, loved ones, and society around them, but also for their own safety in life at home. After war, peacebuilding begins–oftentimes set with conditions from the international community [25]. Yet, while women’s movements often play an imperative role for bringing the warring parties to peace negotiations, women are rarely included at this table, nor are they part of designing what peacebuilding initiatives should be implemented, or how this should be done. This, despite research showing the fundamental importance of bringing in different voices to negotiations, for lasting peace [26].

So, how are women affected by peacebuilding initiatives after war? How do they perceive such initiatives for peace? And do women’s views differ from the views of men? Without asking, we do not know; so we did, in Sri Lanka and Nepal–two countries with clear gendered patterns in both war and peacebuilding. In this article, we present what we found.

Based on previous research, we propose that due to lingering insecurity in the post-war period as described above, women in post-war contexts may hold more negative views than men concerning peacebuilding measures that affect *security in everyday life*. In contrast, we expect no gender differences in attitudes concerning *national elite-level processes* for peacebuilding. Using new survey data collected in Sri Lanka and Nepal, we investigate these interconnections between gender and post-conflict attitudes. Our results support our proposition: while support in general is high for peacebuilding measures among all respondents, women are significantly more negative than men towards those peacebuilding measures that involve increased risk in everyday life, such as truth-telling initiatives and the reintegration of ex-combatants. There are no gender differences pertaining to peacebuilding initiatives that take place far away at the national level, for example, concerning accountability or, in the case of Nepal, the peace agreement.

This article will proceed as follows. We begin by introducing the theory that underpins our study. Then, to situate our work, we give a short account of the armed conflicts in Sri Lanka and in Nepal respectively, and delineate post-conflict challenges for women in each country. Next, we describe the ethical reflections that guided this project, and the formal and informal ethics procedures we followed. The methods of our study are then introduced: how we conducted the survey, developed the questionnaires, designed the sampling, and so on. Thereafter, we present our results. We end with a conclusion, and suggesting some important next steps for future peacebuilding research and policy.

## Theoretical framework

Violence against women is known to increase heavily during and after war [23, 24, 27–29]; also, the types of violence women are subjected to in war often bring stigma after the war has ended [12, 30, 31]. Yet, security for women is generally not a post-conflict political priority. On the contrary, the international norms for peacebuilding design often involve unintended negative side effects for women. For example, truth telling processes have come to play a pivotal role in postconflict peacebuilding around the world; indeed, they are part and parcel of the international community’s post-conflict toolkit, becoming a box to tick for post-conflict countries [9, 32]. The underlying assumption is that truth telling can be healing for society after war and thereby lead to peace [33], and may create a space for a national conversation after which no one can say “I did not know”, or deny the realities of what took place [34]. Similarly, ex-combatant reintegration is often considered important for peace. And, due to financial and other strategic incentives, pressures on post-conflict countries to quickly embark upon these types of transitional justice (TJ) processes after war are high [32, 35]. However, post-conflict regimes may well initiate truth telling and other transitional justice processes to *maintain* power and authoritarianism, for steering *who* is heard and thereby (mis-) directing truth, remembering and forgetting, and for perpetuating violence–a process Loyle and Davenport call transitional *in*justice [36]. Or, post-conflict governments may hurry to embark upon these processes simply–and understandably–to receive desparately needed funds in a shattered post-war economy.

Yet, whatever the reasoning behind these multifacteted calculations for initiating peacebuilding processes after war, the realities of women are often forgotten or neglected–both by domestic governments, and by the international community. Recent research sheds important light on the intersection of peacebuilding, everyday life and gender. Ketola argues for the demonstrated agency of female ex-fighters in Nepal, in making the active decision *not* to engage in politics after the war [37]. Yadav [38] writes how the Nepal war transformed the gendered social landscape for some women; how undertaking previously masculine roles (e.g. as combatants) led to empowerment and insight of discriminatory practices they no longer accepted after the war. However, gender norms are sticky and resilient, and social transformation takes time [39]. If not designed with meticulous care, truth telling after war involves risks, for example of stigma, insecurity, and revenge–oftentimes more so for women due to the types of violence they were subjected to during the war [12]. Indeed, for some, silence can be unspoken communication, and a chosen strategy for coping [40, 41]. Ex-combatants returning to the village or neighborhood may be former perpetrators, representing an additional burden of threat in the community for women [9]. These returning ex-combatants can be men or women, but in either case, military trained, traumatized ex-combatants can exert an intimidating presence and pose a threat to all women. As post-conflict settings inherently entail a high proportion of female-headed households due to the gendered patterns of war [42], such additional risk accentuates an already precarious situation.

This overarching factor of insecurity for women in everyday life after war links to another important dimension, namely psychological ill-health. Studies consistently show that women demonstrate higher levels of psychological ill-health after war than men [8, 43–46]. For example, in a study on trauma after the 2004 tsunami in Sri Lanka, Gunaratne and colleagues [47] found that women, and in particular women who lost family members, showed higher levels of trauma-related symptoms than men. Research also shows that peri-traumatic events–that is, what happens during and after trauma–affects the degree to which post-trauma pathology develops [48]. After war, many women are left widowed. Globally, the predicaments of war widows have been shown to be dire due to the post-war insecurities, economic hardship, and gender discriminating norms and traditions they face [39].

At the same time, research shows that peace does not mean the end of violence for women, in fact the complete opposite: domestic violence increases both during and *after* war. Globally, nearly one in three women experience intimate partner violence (IPV) in their lifetime [49]. In a large multi-country study on male perpetration of intimate violence in the Asia and Pacific region, Fulu et al. found that the prevalence of physical or sexual IPV perpetration is staggeringly high, varying from 25% to 80% [50]. Zooming in on studies on IPV in conflict settings we observe the following. In a population-based survey with 2,244 women in South Sudan, Ellsberg et al. [24] found that between 50% to 65% of women reported having experienced physical or sexual IPV. In Peru, women exposed to conflict violence during the 1980–2000 civil war reported higher levels of partner abuse than women in areas less affected by the violence [51]. Østby [52] found a similar effect in Sub-Saharan Africa, where women living in regions with higher conflict intensity were more likely to be sexually abused by their partner. In a study in the occupied Palestinian territory with 3500 married women, Clark and colleagues found a significantly increased risk of IPV during and after political violence [23]. In the 2006 conflict between Lebanon and Israel, Usta and colleagues found that “one of the most traumatic experiences that women reported during and after the war was dealing with their husbands” [53]. In Afghanistan, where conflict and insecurity has been ongoing for over four decades, Corboz et al. [54] report that the national prevalence of physical, emotional or sexual violence against married women aged 15 to 49 is at 56%.

Such continued everyday threat and trauma creates different challenges for women and men in the post-conflict phase, circumstances that need to begin informing peacebuilding practice. Indeed, based on their study above, Ellsberg et al. [24] conclude that “policies and laws to prevent violence against women and access to justice should be given high priority within the ongoing peacebuilding process in South Sudan”. Due to these extreme circumstances for women, we propose that women in post-conflict societies will hold more negative attitudes towards aspects of peacebuilding that affect everyday life, for (at least) two reasons:

First, at the community level, post-conflict peacebuilding initiatives often focus on issues such as truth telling and the reintegration of combatants–issues that in fact may, unintentionally, involve further risks for women. At the micro level, within families, there is often a re-traditionalization of gender roles in the aftermath of conflict. An increase in domestic violence is often a consequence [24, 29, 51, 52, 54, 55]. At the same time, war widows and female-headed households often face threats and insecurities due to the lack of protection the presence of men represents in many post-conflict societies [39]. This increased risk of insecurity and traumatic exposure in the aftermath of war may lead to more negative attitudes towards peacebuilding measures that may involve yet additional risk, further affecting everyday life among women.

Second, peacebuilding–like war–is gendered. Research shows that the indirect consequences of war, for example, the absence of health care or means for financial survival, affect women disproportionately [11, 30, 39, 56]. In a systematic review, Bwirire et al. [57] illustrate this negative impact of armed conflict on women, and the health inequalities post-conflict settings entail. We argue that the accumulated burden of these negative consequences of war lead to more negative attitudes among women towards peacebuilding initiatives that have a direct effect on their everyday life.

Based on the studies above, we propose that due to the many, accumulated post-war insecurities they face after war, women will hold more negative views than men concerning *peacebuilding measures that affect security in everyday life*. Examples of such measures are locally based truth telling processes, or the reintegration of ex-combatants in the community. In contrast, we expect *no gender differences* in attitudes concerning national elite-level processes. So, for example, we do not expect that attitudes concerning transitional justice processes of human rights abusers will be different between women and men as these processes take place far away, with no particular effect on everyday life. That is, only for peacebuilding efforts that entail a heightened security risk for women at the local, individual level do we expect to find differences in attitudes between women and men.

Based on our first supposition, that initiatives that affect security in everyday life will be perceived as more negative by women than by men, we formulate the following hypotheses.

H1: Women are more skeptical towards coexistance with former enemies.
H2: Women are more skeptical towards truthtelling processes.

Conversely, we do not expect to find a gender gap in peoples’ views on inititatives and processes taking place at the elite level, that does not directly impact on everyday security. Thus, the following hypotheses are formulated:

H3: There is no significant difference between women’s and men’s views on accountability of perpetrators.
H4: There is no significant difference between how women and men view peace agreements.

To investigate these post-conflict inter-connections, we conducted extensive fieldwork in Sri Lanka and Nepal, two countries which have seen multi-faceted efforts to build peace after civil war, but where the peacebuilding processes have been fraught with difficulty. We will now turn to these two countries, to describe our choice of study locations in a bit more detail.

## Our locations of study: Sri Lanka and Nepal

Sri Lanka and Nepal were chosen for this study as gender has played a prominent role both during the war and after the war in each country. In both countries, a significant number of female fighters took part in the war (Sri Lanka: in the LTTE; Nepal: in the Maoist uprising); and peacebuilding has held important gendered dimensions. This latter aspect, however, is true everywhere: post-conflict peacebuilding involves particular challenges for women globally [39, 42]. And of course, at the same time a myriad of differences exist between the wars in the two countries. For example, in Sri Lanka, the war followed ethnic lines, the rebels fought for a separatist state, and the war ended with massive government victory, while in Nepal the groups involved in the armed conflict were mainly organized along ideological lines, fought for government power, and the war ended with a peace agreement. So while every conflict and post-conflict setting is unique in innumerable ways, there are also fundamental similarities–making learnings important for post-conflict settings elsewhere too.

To situate our study, we will now provide short backgrounds to the armed conflicts in Sri Lanka and Nepal respectively, as well as how women have been active in–and affected by–the peacebuilding processes in each country.

### Sri Lanka

#### Sri Lanka’s civil war

Sri Lanka’s civil war (1983–2009), one of the longest wars in South Asia, was fought between the Sri Lanka government’s armed forces and the Liberation Tigers of Tamil Eelam (LTTE) over the formation of a separate Tamil state of Eelam. The rhetoric of homeland and nation that fueled the war meant that it took on ethnic characteristics, pitting a largely Sinhala Buddhist Sri Lankan military which emerged from the majority Sinhala population (74.9%) against a Tamil minority (11.2%) in which Muslims (9.2%) also got caught in the crossfire when the community was expelled from the north and east by the LTTE in its project of achieving an exclusively Tamil homeland.

The numbers dead and missing in the war are a matter of fierce dispute, but it is generally regarded that an approximate 100,000 were killed, with an estimated 7000 dead and 2500 missing in the final five months of the war alone [58]. The war was fiercest in the north and east of the country, the provinces claimed by the LTTE as the Tamil state/homeland. More than a million people living in these regions experienced multiple and protracted displacement [58]. Sixteen thousand cases, moreover, of persons missing in the conflict had been reported by November 2015 to the ICRC [59], while the numbers of enforced disappearances (that could also include those missing in action) were reported at a higher rate of over 20,000 to the Presidential Commission of Inquiry (popularly known as the Paranagama Commission) appointed in 2013 to probe abductions and disappearances in the war [60].

The war ended in a decisive military victory for the Sri Lankan government. As a result, there were no formal negotiations amongst the warring factions on a durable peace through a political settlement and/or a commitment to transitional justice. Sustained efforts at post-war peacebuilding towards better inter-ethnic, civil-military and citizen-state relations remain, even a decade since the war ended, off-target for a number of reasons. The continuing militarization of the north and east, and surveillance of the local population remains a site of anxiety, including for women whose mobility and safety are compromised by regular armed patrols [60]. The use of collaborators and informants by the military for counter-insurgency operations has also bred mistrust within the Tamil community itself, hampering efforts at repair [61]. There has also been little by way of accountability for war crimes, including for conflict related sexual violence (CRSV), and nods to transitional justice mechanisms such as the Office for Missing Persons and the Office for Reparations, established in 2016 and 2018 respectively, remain underfunded, bureaucratized and politicized. Numerous domestic truth-telling initiatives have been created in the 15 years since the war ended, including the 2010 Lessons Learned and Reconciliation Commission (LLRC) and the most recent (January 2024) draft bill for a Commission for Truth, Unity, and Reconciliation. However, these initiatives are critiqued for being government-biased and primarily seeking to deflect international calls for accountability rather than truly grappling with past violations [62, 63]. In the area of post-war reconstruction, the infrastructural and market driven economic development of the north and east which the Sri Lanka government prioritized as a substitute for post-war conflict resolution, including the ending of “separatist tendencies” as former President Mahinda Rajapakse stated, only resulted in the further embitterment of the Tamil population and resentment at the “network of patronage and affluence” that followed [64, 65].

#### Women and peacebuilding in Sri Lanka

The effect of the Sri Lankan armed conflict on women, in particular, was stark. It resulted in an estimated 89,000 war widows who became heads of households, tasked with providing food security and keeping family units together under trying circumstances [39, 66]. An estimated 80% of those internally displaced, moreover, were women [67], and although by December 2018, 97% of IDPs had been resettled [65], re-gaining lost livelihoods in a context of economic devastation and physical and emotional trauma continue to be significant challenges to recovery. As 91% of those reported as missing to the ICRC are male, and over half of the reported number were between 18–29 years of age [59], it is, moreover, their mothers, wives and sisters who continue to deal with the trauma of “ambiguous loss” that disappearances entail, while substituting for lost family income. As victims of CRSV both during the war and at its end, women have also had to survive intimidation and stigma [68]. Many were also disabled in the war, and/or function themselves as caregivers to the disabled within their families. Each of the above points to a disproportionate burden on women who survived the war.

In this context, the assumptions that women are, by nature, more amenable to peacebuilding after conflict, requires revision. In Sri Lanka, women’s needs, entitlements and rights have either been ignored or barely mainstreamed in national policies on post-war reconstruction [39]. For example, there was a Sub-Committee on Gender Issues (SGI) formed during the 2002–2004 peace process in Sri Lanka with the participation of women representatives from the Sri Lanka government, the LTTE, and civil society. These negotiations were sidelined, however, and many of issues raised by the Sub-Committee were not considered in the plenary process [69]. The consequence of these failures, has been the marginalization of women, particularly in the north and east of the country, from access to economic goods, including land and a stable income (many earn a living on a daily wage basis). Many war-affected women are caught in microfinance debt-traps, with loans granted under patriarchal conditions, to pay for a range of expenses including consumption, children’s education and migration overseas of family members from the former war zones [70]. As many of the micro-finance institutions operate in these provinces under license by the Central Bank, replacing traditional sources of lending including NGO-led microfinancing institutions in the community [71], their presence indicates post-war market oriented financial policies that are seemingly about women’s empowerment but in effect, have had a detrimental effect on their post-war recovery and wellbeing.

### Nepal

#### Nepal’s “People’s War”

Nepal’s decade-long armed conflict (1996–2006) was fought between the Maoist rebels and the Government of Nepal. The aim of the Maoist rebels “People’s War” was to abolish the Monarchy and radically transform Nepalese society economically, socially and politically, into a democratic republic [72].

A period of multiparty democracy (1990–1995) had failed to fulfill the expectations of the Nepalese people–high levels of political corruption and deep discrimination and marginalization based on caste, gender, ethnicity, and place of residence remained, leading to nationwide grievances [73–75]. The Maoists used the chaotic political atmosphere of the country to convince marginalised people to wage war against the state [76]. Hence, the Maoist armed struggle in large part originated in accumulated grievances felt by underprivileged people against the Nepalese government [72, 77–79].

The Maoist armed struggle was not considered a serious issue initially; the government treated the armed movement as a problem associated with law and order. However, within five years, the Maoists had expanded into an armed movement with nationwide scope, solid military and political strength, and a strong support-base among marginalised groups [76]. Government security forces, not least the police force, soon lost their capacity to quell the rebel group through military means [80]. In 2000–2001, the armed police force, and later the Royal Nepal Army (RNA), was mobilized against the Maoists. By the time the government paid serious attention to the uprising, the Maoists had developed a strong military capacity, with more than 15,000 combatants and a force capable of challenging the state’s security forces [81].

More than 13,000 people were killed in the 10-year war, thousands abducted or disappeared, and more than 200,000 people were displaced [82]. The toll of the war also included thousands left with injury and trauma, damage to infrastructure amounting to more than two billion US dollars, a stagnated economy, high unemployment, and a serious decline in agricultural production forcing thousands of rural villagers into displacement [77, 83, 84]. Failed peace talks between the Maoists and government in the early 2000’s led to deepened mistrust between the parties [75, 77]. However, political developments in 2005 opened to a peace process. In October 2005, the Seven Party Alliance (SPA), a coalition of seven political parties seeking to end autocratic rule in the country, and the Maoists agreed to the “12-point Understanding”, wherein the parties pledged to work for peace and democracy, and for ending autocratic monarchy. Shortly thereafter, in April 2006, a strong, well-organised nonviolent uprising began through the Nonviolent People’s Movement, becoming a catalyst for the ending of the war [85]. A new, conducive environment for peace negotiations was created, and in November 2006, the Comprehensive Peace Agreement was signed between the Government of Nepal and the Maoists. The agreement proposed two transitional justice bodies, the Truth and Reconciliation Commission (TRC) and the Commission on Investigation of Enforced Disappeared Persons (CIDEP), both of which have faced numerous delays in implementation. Last year, in 2023, Nepal drafted a new bill for Truth and Reconciliation to amend an earlier bill.

#### Women and peacebuilding in Nepal

The armed conflict had a devastating impact on Nepali women. Rape, sexual abuse, torture, and trafficking of Nepalese women were common, committed by both government forces and the Maoists [86–88]. Thousands of women were killed, abducted, disappeared, physically disabled, and made homeless. More than 9,000 women were widowed [89]. When men left to fight or escaped from their home villages, women became head of the household, taking care of children, elderly, and other family members [88]. They were subjected to house raids and interrogation from government security forces on the one hand; feeding and providing shelter to Maoist rebels on the other.

The impact of the armed conflict on women has extended into the post-war period. Women who lost their family members are still struggling for justice. Psychological trauma from the war continues to impact on mental health and wellbeing. The death of male family members means lessened family income and wellbeing. The many young girls who were forced to leave formal schooling due to the closure of schools during war time have faced difficulties in obtaining adequate jobs.

The CPN-M is estimated to have had around 20–40% female combatants (20% according to UNMIN; 40% according to CPN-M). The group espoused a gender equal ideology, and the conflict ended with a negotiated settlement where CPN-M got access to political power-sharing. They thus had every opportunity to focus on empowering women, increase women’s political representation, and consider the agency of women in the design of DDR programs. Yet, this has not happened. Women were not part of the high-level peace negotiation processes. Female fighters have not been granted access to the political gains made by CPN-M, and DDR programs have either excluded women or focused on gender stereotypical tasks. Furthermore, on their return to mainstream society, they were stigmatized for breaking their assigned gender norms and many struggle with their livelihood [88, 90–92].

More broadly, the peace agreement and other negotiated documents have failed egregiously to acknowledge the impacts of war on women [87, 93]. Indeed, much of the conflict-related women’s agenda has remained unaddressed until now. A number of factors have contributed to this situation, for example, rigid patriarchal structures and apathy within political parties to address women’s demands, the lack of formal and specific agreements for addressing the needs of women, weak advocacy from civil society and conflict victim associations, and a lack of pressure on the government from the international community to include women and women’s perspectives in the peacebuilding process.

## Methods

To empirically assess our hypothesis that women will be more negative than men towards peace-building initiatives that directly affect security in daily life, we collected survey data with 2,041 women and men in Sri Lanka and Nepal. In both countries, we also conducted focus groups and key informant interviews but in this article, we focus on the survey data. We will now describe how the surveys were conducted, beginning with the ethical reflections that guided our work. More details about the surveys can be found in the S1 Appendix.

### Ethics

Considering the sensitivity of asking questions on war and peace in post-conflict communities, ethical reflection is an integral part of any peace research project [94]. Our aim in peace research to learn in the hope of making peacebuilding safer and more sustainable for those involved, must constantly be weighed against potential risk for participants. To do no harm overrides all else. In this project, ethical reflection was woven in throughout; indeed, it drove the very research question. Women’s perspectives on how peacebuilding initiatives affect their life are lacking. At heart, knowing how peacebuilding affects and is perceived by women is an integral, ethical part of peace research, as we need to include *all* perspectives to do research that upholds the three basic ethical principles: 1) respect of persons, 2) to maximize benefits and minimize harm, and 3) that people are treated equally [95]. To hear women’s voices is central for both increasing our understanding of peacebuilding, for building peacebuilding interventions that do no harm, and for ethical peace research.

The ethics process guiding this project consisted of two dimensions: *informal* ethics and *formal* ethics. The informal ethical dimension involved holding ethics conversations within our project team, continuously discussing challenges and dilemmas we could foresee or encountered. This included everything from designing the very research project and survey questions, to when to conduct the survey (navigating monsoons and political rallies), where the survey should be conducted and with whom (including as many voices as possible while minimizing potential risk), how to formulate survey questions (and which questions to exclude, to minimize risk of retraumatization) and in what order (to lessen emotional charge), and so on. These conversations intensified in the phase of field work–before, during and after–to hold an eye on potential areas of risk [94]. Important to note too, is that in the field, before beginning the actual survey interview, the enumerator asked the interviewee for their consent to participate, as well as informing them that they could choose to decline or discontinue the interview at any time. The informed consent statement was read verbally to the interviewee, and their response noted in the PDA (ticking of box by the enumerator), after which the survey interview proceeded.

The formal ethics dimension involved following each country’s formal ethics procedures, obtaining ethics approvals at the national level in Sri Lanka, Nepal, and Sweden (Regional Ethics Committee, Dnr 2016/342 and Dnr 2016/342/1) respectively, as well as at all regional levels and local levels in Sri Lanka and Nepal, in accordance with local regulations and customary practice.

### Survey

The survey questionnaire, developed in close collaboration between Uppsala, Kathmandu, and Colombo, has seven sections, asking questions on: demographics, trauma stressors, PTSD symptoms, experiences of family violence, resilience factors, peacebuilding attitudes, and gender equality attitudes. While we aimed to streamline the questions across the two countries, they were also designed and adapted to fit each context (Sri Lanka and Nepal) and modified after piloting for cultural and conflict sensitivity; hence, the inclusion and wording of some questions differed between countries. Additionally, the sampling procedures differed to tailor to the specific circumstances of each country’s conflict background, and ensure our study would be meaningful to each context. It should be noted that neither sample aspires to be nationally representative; hence, the results cannot be generalized to the whole population. However, findings of this study are relevant at least in areas similar to the study districts, and can open to interesting questions for each country as a whole.

In *Sri Lanka*, the survey study was carried out between October 27 to November 15, 2017. In total, 1,028 respondents (50% women, 50% men) were interviewed for the survey. The survey was carried out in three districts to reach populations affected by the Sri Lankan war, while ensuring equal distribution between the three ethnic communities: Anuradhapura (Sinhalese respondents, N: 341), Vavuniya (Tamil respondents, N: 346) and Mannar (Muslim respondents, N: 341). Villages in each district were chosen according to probability proportionate to size (PPS) sampling, and starting points were distributed according to their population proportion.

In *Nepal*, the survey data was collected between 3 February to 3 March, 2018. It included 1,013 respondents (50% women, 50% men) from six districts throughout the country. The six districts were selected and categorized based on conflict intensity, using data on killings, disappearances, and displacement as recorded through the Informal Sector Service Centre (INSEC) and by the Government of Nepal’s categorization of districts based on the intensity and impact of the armed conflict. Three districts are categorized as high conflict areas (HCAs: Bardiya, Surkhet and Dang) and three districts as low conflict areas (LCAs: Sunsari, Morang and Jhapa). Around 60% of respondents in the Nepal study were from HCA districts, and 40% were from LCA districts. In each district, municipalities/villages, wards, and small settlements within each ward were selected randomly, proportional to population size and gender balance.

The survey data was collected using Personal Digital Assistants (PDAs, i.e. tablets) to maximize anonymity and minimize known interviewer-effects such as social desirability bias and acquiescence. However, many respondents opted to have the enumerators assist them in typing in their responses (48% in Sri Lanka; 61% in Nepal).

#### Measurement of key variables

Our main independent variable is *Gender*, coded (1) for female respondents and (0) for male respondents. We expect female respondents to be more skeptical in relation to initiatives that directly influence security in their *daily live*s, while we expect no gender difference in the views of initiatives driven at the *elite-level*, hence a range of different dependent variables are included in the statistical analysis. The two kinds of initiatives (daily life vs elite level) are summarized in Table 1 below.

**Table 1:** Theoretical expectations and measurements.

		Sri Lanka	Nepal
Peacebuilding initiatives affecting daily life: **Gender difference expected**	**Everyday coexistence**	• Trust in Sinhala • Trust in Tamil • Trust in Muslim • Feel threatened around Sinhala • Feel threatened around Tamil • Feel threatened around Muslims • Feel comfortable around Army • Feel comfortable around ex-LTTE	• Trust in Army • Trust in Maoists • Feel threatened around Army • Feel threatened around Maoists • Feel comfortable around Maoists
	**Truthtelling**	• Important to collect testimonies • Sharing truth build positive relationships	• Important to collect testimonies • Sharing truth build positive relationships
Peacebuilding initiatives at elite level: **No gender difference expected**	**Accountability**	• Perpetrators should be held responsible	• Perpetrators should be held responsible
	**Peace accord**	N/A	• The CPA was necessary to end conflict • The CPA reflects the will of the Nepali people

In both surveys, we aimed to ask questions capturing each construct. However, the specific questions included, and in some cases the wording, differ across the two cases for several reasons. First, the type of conflict differed, meaning that for Sri Lanka inter-ethnic relations is relevant to ask about, but less so in Nepal. Second, the conflicts ended in different ways, meaning that in Nepal we ask about perceptions of the Comprehensive Peace Agreement. Third, some questions were too sensitive to ask in one context and not the other, which explains the difference in asking about coexistence and reintegration.

*Everyday coexistence* intends to capture issues around interacting with former combatants with a sense of trust and without fear. To this end, we include three commonly used co-existence measures in peacebuilding research: how much respondents **trust** members of former armed groups and other ethnic groups, to what extent respondents **feel comfortable** around them, and to what extent respondents **feel threatened** around them. The fundamental importance of trust for peace has been recognized for a long time in the scholarly literature [96–98]. Research shows that through even the smallest glimmers of trusting relations in conflictual settings–maybe through just one or two well-respected people with contacts on the ‘other’ side–societal, informational networks can begin to be built across divides, decreasing rumours of threat and slowly increasing trust and relationships [99]. To study willingness for co-existence, many survey research projects ask–as did ours–how comfortable people feel in different situations, for example in sharing a meal with a former enemy or ex-combatant [100]. That is, instead of asking people directly what they think of the former enemy or of reintegrating ex-combatants–which may often be highly sensitive and difficult to answer–one asks around. The actors and groups that we ask respondents to think of in terms of e.g. trust and threat perceptions naturally differ across the two cases. In the case of Sri Lanka, we ask about other ethnic groups, while in Nepal we asked about specific armed groups. In line with our theory, we expect that women will be more skeptical than men regarding these everyday coexistence measures.

*Truthtelling*–the second everday-life measure–relates both to how respondents view institutional mechanisms for collecting testimonies and sharing the truth about past atrocities (such as truth commissions) and if talking about the past in everyday life has positive effects. Here again, we expect that women will be more skeptical than men.

We then have the two elite-level peacebuilding initiative categories. *Accountability* refers to how respondents view processes whereby perpetrators of violence during the armed conflict are held responsible for their crimes–regarding this question we expect no gender difference. *Peace accord* relates to how respondents (in Nepal only) view the peace agreement, again with no expected gender difference.

All questions included in the survey and analyzed for the present study are described further in the S1 Appendix.

The following variables were included as control variables that could potentially mediate the relationship between gender and peacebuilding attitudes. First, *Affected by conflict*, measures the extent to which respondents or their families were exposed to, for example, violence, displacement, or attacks during the conflict. Second, a binary measure for symptoms of Post-traumatic Stress Disorder (*PTSD*) is included. While assessing PTSD across cultures is tricky–traumatic experience is spoken of (or not spoken of) in different ways in different settings–it can give an indication of the magnitude of war-related trauma in a population, and its accompanying difficulties after war. To minimize exposure to difficult memories, we asked the short 6-question measure of PTSD previously used in war-affected settings, using the same cut-off rates (0 = no PTSD; 1 = PTSD) [101]. Third, we include a measure for *Physical health*. Fourth, a dichotomous variable for the respondents’ *Education* is included, coded (0) for those with no formal school or only primary school (Nepal) or between grades 1–5 (Sri Lanka); those with secondary school or higher (Nepal) or grades 6–11 or higher (Sri Lanka) are coded (1). Fifth, we control for the *Family finance* where respondents report how they feel about their current financial situation, ranging from very hard to very good. Last, for the analyses of Nepal, we control for whether or not the respondent lives in a High conflict area, whereas for Sri Lanka ethnicity dummies are included (Muslim and Tamil; Sinhalese as reference category).

Ordered logistic regression models were estimated for Likert scaled dependent variables and OLS regression were used for continuous dependent variables.

## Results

We begin with some descriptive background–who did we talk with? In *Sri Lanka*, a total of 1,028 people were interviewed in the survey from three districts: Vavuniya, Mannar, and Anuradhapura (50% women/50% men in each district). 98% of our respondents resided in Sri Lanka during the conflict. Many had been exposed to violence and other conflict-related experiences, for example, over 70% had been displaced at least once during the war and 33% were exposed to violence. A staggering 42% (44% of women; 39% of men) met the criteria for PTSD. This is extraordinarily high compared to non-war settings where levels of PTSD range from 0,5%-2,5% in Western European countries [102] to 7% in the USA [103], and also to post-war settings that experienced lower levels of violence during the war than Nepal, e.g. Solomon Islands, around 15%; [9]. Further, 72% of the Sri Lankan respondents reported that their physical health was “somewhat good” or “very good” while 28% reported “somewhat poor” or “very poor”. Regarding education, 79% of the respondents had secondary or higher levels of education, while 21% had no formal school or up to grade five. Last, regarding the family’s financial situation, 63% reported that the situation was “very hard” or “a little hard”, while the remaining 37% responded “good” or “very good”. Additionally, many (25%) also responded “struggle for economic wellbeing” when asked what they consider their greatest current threat (to compare with e.g 5% for “renewed violence in my community”).

In *Nepal*, a total of 1,013 people were intervieweed in the survey from six districts (50% women and 50% men in each district). Almost all (96%) resided in Nepal during the conflict. 12% reported that they were displaced during the conflict and 26% experienced some form of violence. Nearly one-third of the Nepali respondents, 32%, reached the threshold for PTSD (no significant gender difference); again shockingly high compared to non-war settings. In Nepal, 72% reported that their physical health was “somewhat good” or “very good” while 28% reported “somewhat poor” or “very poor”. Regarding education, 54% of the respondents had secondary or higher levels of education, while 46% had no formal school or only primary school. Last, regarding the family’s financial situation, 37% reported that the situation was “very hard” or “a little hard”, while the remaining 63% responded “good” or “very good”. Also in Nepal, many respondents (14%) listed “struggle for economic wellbeing” first when asked what they consider their greatest current threat.

Our aim with presenting these descriptives is not to compare the two countries, but to present both post-conflict settings in their own right. What is interesting to this study, is to investigate to what extent we find similar patterns in women’s and men’s attitudes to peacebuilding, in two very different post-conflict contexts.

We now move on to our analyses on gender to test the hypotheses. We proposed in the theory section above, that due to the many, accumulated post-war insecurities they face after war, women will hold more negative views than men concerning *peacebuilding measures that affect security in everyday life*, such as locally based truth telling processes, or the reintegration of ex-combatants in the community. We expect *no gender differences* in attitudes concerning national elite-level processes, such as transitional justice processes of human rights abusers, as these processes take place far away, with no particular effect on everyday life. That is, only for peacebuilding efforts that entail a heightened security risk for women at the local, individual level do we expect to find differences in attitudes between women and men.

The S1 Appendix contains frequency data on the distribution of all dependent variables, by gender. The S1 Appendix also reports a number of alternative estimations and robustness tests. In the analysis presented below, post-estimations to provide substantial interpretations of coefficients are presented when results show significance.

Based on our first supposition, that initiatives that affect security in everyday life will be perceived as more negative by women than by men, our first hypothesis reads:

H1: Women are more skeptical towards coexistance with former enemies.

To assess H1, we will look at measures of coexistence and trust; we begin by examining Sri Lanka.

Table 2 shows the results from the regression analysis (ordinal logit in Models 1–6 and OLS in Modesl 7–8). We include questions about trust (Models 1–3) and threat (Models 4–6) in relation to other ethnic groups in Sri Lanka, as well as how comfortable respondents feel around members of the army and former LTTE cadres (Models 7–8). We subset the analysis so that questions about e.g. trust in Sinhala is analyzed only for non-Sinhala respondents (Tamils and Muslims) etc.

**Table 2:** Coexistence and trust Sri Lanka.

	(1)	(2)	(3)	(4)	(5)	(6)	(7)	(8)
	Trust Sinhala	Trust Tamil	Trust Muslim	Threat Sinhala	Threat Tamil	Threat Muslim	Comfortable Army	Comfortable ex-LTTE
female	-0.218	-0.553***	-0.529***	0.142	0.058	0.067	-0.722***	-0.485**
	(0.172)	(0.174)	(0.179)	(0.156)	(0.164)	(0.168)	(0.207)	(0.231)
affected	-0.029	0.042	0.006	0.096***	0.016	0.010	-0.070*	0.104**
	(0.026)	(0.028)	(0.027)	(0.025)	(0.027)	(0.026)	(0.036)	(0.040)
ptsd14	-0.004	0.388**	0.432**	0.080	0.072	0.226	0.179	-0.097
	(0.178)	(0.188)	(0.203)	(0.162)	(0.179)	(0.189)	(0.232)	(0.258)
physhealth	-0.104	0.029	0.008	-0.083	0.077	-0.005	-0.081	-0.022
	(0.097)	(0.097)	(0.092)	(0.090)	(0.094)	(0.088)	(0.115)	(0.129)
education	-0.430*	0.114	0.033	0.444**	-0.127	0.074	-0.016	0.542*
	(0.235)	(0.227)	(0.236)	(0.206)	(0.212)	(0.225)	(0.272)	(0.302)
famfinance	-0.010	0.117	0.050	0.227**	0.097	0.046	0.080	0.126
	(0.115)	(0.112)	(0.115)	(0.104)	(0.106)	(0.109)	(0.136)	(0.152)
muslim							-5.499***	-0.386
							(0.280)	(0.313)
tamil							-6.373***	3.331***
							(0.292)	(0.327)
/cut1	-2.027***	-0.554	-0.475	0.698*	0.330	0.085		
	(0.456)	(0.416)	(0.406)	(0.415)	(0.397)	(0.380)		
/cut2	0.900**	2.374***	2.808***	2.537***	2.635***	2.128***		
	(0.447)	(0.431)	(0.436)	(0.428)	(0.416)	(0.393)		
Constant							10.617***	1.946***
							(0.497)	(0.551)
Observations	549	554	523	590	570	535	989	987
R-squared							0.459	0.207

Standard errors in parentheses; *** p<0.01

** p<0.05

* p<0.1

The results indicate that while women in Sri Lanka report lower levels of inter-ethnic trust (Models 1–3), there is no gender difference in perceived levels of threat (Models 4–6). Examining the frequency distributions of trust and threat (for details, see S1 Appendix), we can see that inter-ethnic trust is quite low and threat perceptions are relatively high. Moreover, threat perceptions vary: non-Sinhala respondents feel relatively threatened around Sinhala while non-Tamils feel less threatened by Tamils, and non-Muslims less threatened by Muslims.

In substantial terms, Model 2 (Trust Tamil) post-estimations show that the predicted probability that women in Sri Lanka respond *Not at all* is 0.32 compared to men, 0.19; the corresponding numbers for *A little* is 0.60 and 0.66 and *Very much* 0.08 compared to 0.15. The gender gap is similar in Model 3 (Trust Muslim), and not significant in Model 1 (Trust Sinhala).

Furthermore, Models 7–8 (ex-combatants) show that women are significantly less comfortable then men around both ex-LTTE and the army. The dummy variables for ethnic groups also report results in line with expections: each group has the most trust in their own group. Furthermore, Muslims and Tamils trust each other more than they trust Sinhala. In separate analyses not reported above (see S1 Appendix), we also find that women report lower levels of generalized trust (i.e. without reference to specific groups).

In Table 3, we continue to explore H1—how respondents feel about coexisting and trust—now in Nepal. The Nepal survey includes questions about perceived level of trust, and of feeling threatened by, both Maoists and members of the army (Models 1–4). It also includes a question about the extent to which respondents feel comfortable around Maoists (Model 5).

**Table 3:** Coexistence and trust Nepal.

	(1)	(2)	(3)	(4)	(5)
	Trust Maoists	Trust Army	Threat Maoists	Threat Army	Comfortable Maoists
female	-0.086	-0.304**	0.812***	0.796***	-1.041***
	(0.131)	(0.131)	(0.139)	(0.138)	(0.295)
affected	0.061**	-0.065**	0.006	0.069**	0.155***
	(0.026)	(0.026)	(0.028)	(0.027)	(0.059)
PTSD	0.073	0.114	0.337**	0.248*	-0.418
	(0.148)	(0.145)	(0.150)	(0.149)	(0.328)
physhealth	-0.055	-0.024	0.194**	0.072	-0.044
	(0.072)	(0.073)	(0.078)	(0.075)	(0.162)
education	-0.045	0.434***	0.342**	0.354***	-0.923***
	(0.132)	(0.131)	(0.138)	(0.137)	(0.297)
famfinance	-0.001	0.163*	0.067	-0.086	-0.171
	(0.093)	(0.093)	(0.098)	(0.097)	(0.207)
highconflict	-0.058	-0.154	0.420***	0.408***	-0.464
	(0.133)	(0.132)	(0.141)	(0.139)	(0.300)
/cut1	-0.315	-0.774**	2.089***	1.361***	
	(0.328)	(0.328)	(0.361)	(0.346)	
/cut2	1.947***	1.663***	3.821***	3.317***	
	(0.337)	(0.333)	(0.378)	(0.363)	
Constant					10.707***
					(0.729)
N	902	921	917	932	971
R-squared					0.034

Standard errors in parentheses; *** p<0.01

** p<0.05

* p<0.1

As shown in Table 3, women generally feel more threatened both by Maoists and by the army compared to men. They also have less trust in the army and feel less comfortable around Maoists. There is no significant gender difference in trust in Maoists (Model 1). Frequency distributions (see S1 Appendix) show that while most Nepali respondents have low trust in both the Maoists and the Army, luckily, most do not feel threatened by them.

In substantial terms, holding other variables at mean values, the results from Model 2 (Trust Army) indicate that the predicted probability of not trusting the army at all (*Not at all*) is 0.29 for women and 0.23 for men. The predicted probabilities for *A little* are 0.53 for women and 0.54 for men, and for the highest category (*Very much*), the predicted probabilities are 0.17 for women and 0.22 for men. Hence overall, women in Nepal have lower trust in the army than men.

For Model 3 (Threat Maoist), the predicted probability of responding *Not at all* is 0.49 for women and 0.69 for men; predicted probabilities of *A little* are 0.35 for women and 0.24 for men, and for the highest category (*Very much*), the predicted probabilities are 0.16 for women and 0.07 for men. In other words, here we see a quite a large gender gap, wherein women feel more threatened by Maoists than men. Model 4 also shows that female respondents feel more threatened by the army. The predicted probabilities of responding *Not at all* on perceived threat is 0.49 for women and 0.68 for men; responding *A little* is 0.38 for women and 0.26 for men, and *Very much* is 0.13 for women and 0.06 for men.

Model 5 (based on OLS regression where the dependent variable is measured on a scale fron 0 to 15); shows that female respondents feel less comfortable than male respondents around Maoists.

In additional regressions not reported above (see S1 Appendix), we also tested a measure for generalized trust (i.e. without reference to specific groups). The pattern remains: women report significantly less trust than men.

In sum, the results from Tables 2 and 3 partially support our expectations. Women in both countries have less trust and feel more uncomfortable towards conflict actors compared to men. The pattern is found in both contexts (Sri Lanka and Nepal) and in relation to both types of conflict actors (former rebels and soldiers from the army). In Sri Lanka we focus on interethnic trust and threat perceptions and the results suggests women have lower trust. Perceived threat by other ethnic groups is low among all respondents, with no gender difference. In Nepal, respondents were asked about trust and threats in relation to armed groups (Maoists and Army): women feel more threatened than men both by Maoists and by the army. Hence, former combattants were perceived as threatening for women in Nepal more than a decade after the war. Given the differences in measures for trust and threat perceptions across the two cases, the results are not comparable across the two cases. However, the findings are revealing and relevant for assessing overall general perceptions of everyday coexistence in each case.

Next, in Table 4, we assess H2 that proposes a gender gap in people’s views on truthtelling in various forms:

**Table 4:** Truthtelling Sri Lanka and Nepal.

	Sri Lanka		Nepal	
	(1)	(2)	(3)	(4)
	Collecting testimonies	Sharing truth	Collect testimonies	Sharing truth
female	0.127	-0.282**	-0.431***	-0.158
	(0.124)	(0.123)	(0.149)	(0.141)
affected	0.065***	0.010	-0.012	0.025
	(0.022)	(0.021)	(0.030)	(0.029)
PTSD	-0.042	0.004	0.223	0.211
	(0.140)	(0.137)	(0.169)	(0.156)
physhealth	0.090	0.084	-0.202**	-0.119
	(0.069)	(0.069)	(0.086)	(0.080)
education	0.117	0.231	0.493***	0.142
	(0.162)	(0.160)	(0.149)	(0.143)
famfinance	-0.004	-0.045	-0.248**	-0.133
	(0.082)	(0.081)	(0.110)	(0.105)
muslim	-0.293*	0.000		
	(0.168)	(0.168)		
tamil	0.302*	-0.283		
	(0.174)	(0.172)		
highconflict			0.213	-0.199
			(0.152)	(0.147)
/cut1	-1.069***	-1.888***	-3.615***	-3.631***
	(0.301)	(0.302)	(0.416)	(0.398)
/cut2	-0.581*	-1.372***	-3.130***	-3.049***
	(0.298)	(0.297)	(0.409)	(0.386)
/cut3	0.814***	0.328	-1.880***	-1.260***
	(0.298)	(0.293)	(0.398)	(0.371)
N	946	955	901	881
R-squared				

Standard errors in parentheses; *** p<0.01

** p<0.05

* p<0.1

H2: Women are more skeptical towards truthtelling processes.

In both the Sri Lanka and Nepal questionnaires, two questions aim to capture these views. Model 1 and 3 reports the results regarding the respondents’ views in collecting testimonies about the conflict and Model 2 and 4 whether they think sharing the truth creates positive relationships (Table 4).

As can be seen in Table 4, the results are mixed. In Sri Lanka, we find no significant gender difference in terms of collecting testimonies, but women are significantly more negative than men in terms of sharing the truth. Substantially (post-estimations based on Model 2) we find that the predicted probabilities to S*trongly disagree* are 0.13 for women and 0.10 for men and to S*trongly agree* the corresponding probabilities are 0.42 and 0.49.

In Nepal, the results are the other way around. Women are more skeptical than men regarding the question about collecting testimonies. On the other hand, there is no gender difference in terms of sharing the truth. Post-estimations of Model 3 (collecting testimonies), suggest that the predicted probability that female respondents *Strongly disagree* is 0.08 and for male respondents 0.06. On the other extreme, female respondents are predicted to *Strongly agree* at 0.66 while male respondents 0.75. In other words, men are 15% more likely than women to strongly agree that collecting testimonies is important.

To understand these data better, we investigated the descriptive statistics (please see S1 Appendix). This inquiry importantly revealed that support for truth telling is high (both ‘collecting testimonies’ and ‘sharing the truth’) among both women and men, in both Sri Lanka and Nepal. Hence, truthtelling is seen as an important peacebuilding measure. However, there are gendered variations: in Nepal, women are more reluctant to collecting testimonies from the conflict than men, in Sri Lanka, women are more skeptical to talk about the past than men. Our survey data cannot tell us, but this skepticism among women could be linked to the importance of silences as mentioned above [40, 41]. In Sri Lanka, for example, Höglund [104] argues that women testifying to the LLRC used silence on particular topics, namely ongoing insecurities and sexual violence, as a strategy to mitigate risk of truth-telling admist a broader context of a victor’s peace.

The second part of our theoretical framework suggests that there will be no difference between women and men in their views on peacebuilding initiatives and processes taking place at the elite-level, far away from daily life. H3 suggests this is the case with regards to accountability of perpetrators:

H3: There is no significant difference between women’s and men’s views on accountability of perpetrators.

In Table 5, the results relating to H3 from both Sri Lanka and Nepal are presented.

**Table 5:** Accountability Sri Lanka and Nepal.

	(1)	(2)
	Sri Lanka	Nepal
female	-0.040	-0.148
	(0.136)	(0.149)
affected	0.049**	-0.022
	(0.025)	(0.029)
PTSD	-0.099	0.130
	(0.152)	(0.166)
physhealth	-0.009	-0.205**
	(0.076)	(0.085)
education	0.478***	-0.194
	(0.174)	(0.152)
famfinance	0.098	-0.098
	(0.089)	(0.108)
muslim	0.636***	
	(0.185)	
tamil	0.790***	
	(0.192)	
highconflict		-0.232
		(0.155)
/cut1	-1.208***	-3.666***
	(0.327)	(0.409)
/cut2	-0.645**	-3.135***
	(0.322)	(0.402)
/cut3	0.627*	-1.983***
	(0.321)	(0.392)
Observations	942	863

Standard errors in parentheses; *** p<0.01

** p<0.05

* p<0.1

We see in Table 5, that the results are in line with the theoretical expectation: there is no gender difference in how respondents view accountability of perpetrators. Among both women and men, support for accountability is high; the descriptive statistics (please see S1 Appendix) show that a majority think perpetrators should be held accountable: in Sri Lanka, 86% agree somewhat or completely to this statement, in Nepal 87%. We suggested in our theoretical framework, that we would see no gender difference because accountability processes do not directly influence people’s [women’s] lives, and are not typically linked to their security situation–our findings would support this claim.

Our final hypothesis, H4, suggests that there will not be a gender gap in how women and men view peace agreements:

H4: There is no significant difference between how women and men view peace agreements.

This hypothesis is only assessed with regards to Nepal and the Comprehensive Peace Agreement (CPA) signed in 2006 (as the war in Sri Lanka ended with victory for the Government, not through a peace agreement). Respondents were asked whether the CPA was necessary to end the conflict (Model 1) and whether it reflects the will of Nepali people (Model 2).

In line with the theoretical expectation, there is no evidence of a gender difference in the respondents’ views of the peace agreement (Table 6). Descriptive data indicates high support for the agreement (no gender difference) and that a large majority agree that the peace accord reflects the will of the Nepali people. When interpreting these results it should be noted that respondents were also asked the question “Have you heard about the Comprehensive Peace Accord 2006-Bistrit Shanti Samjhauta”, and only 43% of the respondents (29% of the women; 57% of the men) answered affirmatively. Only those that answered affirmatively were prompted the following questions about what they think about the CPA (whether the CPA was necessary to end conflict and whether if reflects the will of the Nepali people), hence both significantly reducing and skewing the sample.

**Table 6:** Peace accord views Nepal.

	(1)	(2)
	CPA necessary	CPA reflects Nepalis
female	-0.009	-0.121
	(0.339)	(0.230)
affected	-0.072	-0.006
	(0.055)	(0.041)
PTSD	-0.032	-0.197
	(0.354)	(0.238)
physhealth	-0.421**	-0.229*
	(0.202)	(0.130)
education	0.418	-0.216
	(0.339)	(0.251)
famfinance	-0.130	-0.298*
	(0.245)	(0.167)
highconflict	-0.009	0.119
	(0.342)	(0.231)
/cut1	-5.721***	-5.349***
	(0.999)	(0.693)
/cut2	-5.170***	-4.582***
	(0.968)	(0.658)
/cut3	-3.678***	-2.525***
	(0.932)	(0.623)
N	422	414

Table 7 below returns to the theoretical framework and summarizes the main results from the empirical analysis.

**Table 7:** Summary of results.

		Sri Lanka	Nepal
Peacebuilding initiatives affecting daily life: **Gender difference expected**	Everyday coexistence	Mostly supported	Supported
	Truthtelling	Partially supported	Partially supported
Peacebuilding initiatives at elite level: **No gender difference expected**	Accountability	Supported	Supported
	Peace accord	N/A	Supported

## Conclusions

To better understand the interconnections between gender and post-conflict attitudes to peacebuilding, we asked 2,041 women and men in Sri Lanka and Nepal of their views on post-war peace initiatives. Despite evidence of insecurity and risk for women in peacebuilding after war, little has been known of women’s attitudes towards these processes. Women play key roles in and for peacebuilding, yet seldom have a formal place at the table in peace negotiations or in the drafting of peace agreements [105]. The prevailing notion seems to have been that there is no reason to ask women: previous work in the Global North shows that women are more prone to peacebuilding than men. However, this ‘women and peace’-hypothesis was not supported in two previous studies in post-war developing settings in the Global South: Rwanda and Solomon Islands [8, 9]. Inspired by these findings we set out to explore the relationship between gender and peacebuilding by suggesting specific conditions and causal pathways under which we expect to find a gender gap and move the empirical focus to Sri Lanka and Nepal, two countries with clear gendered patterns in both their wars, and their peacebuilding processes.

Among women and men in both countries, support for everyday/local and elite-level/national peacebuilding initiatives is high. Yet, in line with our theoretical expectations, we find that women are significantly more negative than men towards peacebuilding measures that involve increased risk in everyday life, such as truth-telling and coexistence with ex-combatants reintegrating into the communities. There are no gender differences pertaining to peacebuilding initiatives that take place far away at the national level, for example, concerning accountability or, in the case of Nepal, the peace agreement.

Based on these findings, we suggest that women’s skepticism towards post-war initiatives for peacebuilding that have potential effects on everyday security (in this study defined as truth-telling processes and ex-combatant reintegration) may be more universal than previously known. We propose that this may be caused by the circumstance that some aspects of the global peacebuilding architecture lead to similar experiences for women in diverse post-conflict settings–burdens of which the international peacebuilding community are not fully aware. The skepticism or reluctance among women to certain aspects of the peacebuilding architecture as seen in our study, could be seen as a form of postwar agency [37], of expressing empowered protest [38], In this study, we found the same pattern in both Sri Lanka and Nepal, despite the many stark differences that exist between these two post-conflict settings. With this article, our aim is to increase our understanding of the challenges women face after war, in order to contribute to better, evidence-based peacebuilding practice–of benefit to both men *and* women. Future research would do well in investigating these and other gendered dimensions of peacebuilding initiatives further, in other post-conflict settings, too. Also, how spaces for *silences* could be incorporated in settings where truth telling entails risk, and understanding better what silences represent for postwar peacebuilding needs to be carefully studied [40, 41]. While research takes time, the international peacebuilding community can begin directly by listen carefully to local expertise of all genders when designing peacebuilding interventions.

The data collection for this study took place in 2017/2018; important developments have taken place in both countries since then. For example, the war might seem more distant today and other factors–not least the 2022 financial and political crisis in Sri Lanka–more in the foreground. Yet, our data presents a unique snapshot of women’s and men’s views on peacebuilding initatives, several years after the ending of war. Increased knowledge of the dynamics and challenges of peacebuilding initiatives is of urgent importance: not least for understanding–and being able to prevent–subsequent political, financial and societal crises, that are often instrinsically linked to the war. This spiral of conflict–failed development–more conflict is known as the “conflict trap” [106]. With this article, we contribute to the emerging literature on the microdynamics of peacebuilding [39].

Before ending, we should also mention the proviso that our findings in no way mean that women would be against peace, or not work for peace. A quick empirical glance across the globe would immediately refute such a notion–to the contrary, it would demonstrate that an abundance of peacebuilding work is initiated and led by women around the world [107]. The aim of this article is instead to shed light on the challenges and circumstances under which peace is built. By identifying areas of difficulty, we may find ways to mitigate unknown and unintended challenges, and thereby improve the prospects for peace for both men and women.

## Supporting information

S1 File Information about replication data, do-files, and codebooks. (DOCX)

S1 Questionnaire Sri Lanka gender and peacebuilding 2017. (PDF)

S2 Questionnaire Nepal gender and peacebuilding 2018. (PDF)

S1 Appendix (PDF)

S1 Data (DTA)

S2 Data (DTA)

S3 Data (DO)

S4 Data (DO)
