# Supplementary material for: Women, peace and insecurity: The risks of peacebuilding in everyday life for women in Sri Lanka and Nepal
Source: PLoS One. 2024 May 29;19(5):e0303023. doi: 10.1371/journal.pone.0303023 (PMC11135728; doi:10.1371/journal.pone.0303023)
Supplement: S1 File — (DOCX) [file pone.0303023.s001.docx]

**Information about replication data, do-files, and codebooks**

The replication files and supporting information for the article Brounéus et al (2024) Women, Peace and Insecurity: The risks of peacebuilding in everyday life for women in Sri Lanka and Nepal, *PlosOne* contain the following:

Sri Lanka

Data: sri_lanka.dta

Do-file: survey_sri_lanka.do

Nepal

Data: nepal.dta

Do-file: survey_nepal.do

Appendix

File: online_appendix.pdf

Summarizes the survey information, variable measurements, descriptive statistics, and robustness tests.

Questionnaires/codebooks

The full questionnaires (one for Sri Lanka, one for Nepal) are available in the replication files. Some of the variables described in the questionnaires have been excluded from the replication files, either for ethical reasons (identifying information e.g. household number, longitude/latitude, IPnumber, respondentID) or because they were not used for the present study and hence not necessary for replication purposes (e.g. questions about gender norms, experiences of domestic violence).

Many variables have been recoded for the statistical analysis, such as by creating dummies, reversing scales to facilitate interpretation, or combining variables. These variable transformations are included in the first part of the do-files, under the heading Data preparation.

*Dropped Sri Lanka*

StartDate EndDate interview_length IPAddress RecordedDate ResponseId LocationLatitude LocationLongitude UserLanguage Q1 Q6 Q8 Q21 Q22 Q23 Q25 Q26 Q27 Q29 Q31 Q38_1 Q38_2 Q38_3 Q38_4 Q38_5 Q38_6 Q38_7 Q38_8 Q39 Q40_1 Q40_2 Q40_3 Q40_4 Q40_5 Q40_6 Q40_7 Q40_8 Q40_10 Q41_1 Q41_2 Q41_3 Q41_5 Q42_1 Q42_2 Q42_3 Q42_4 Q42_5 Q42_6 Q42_7 Q42_8 Q44 Q45_1 Q45_2 Q45_3 Q45_4 Q45_5 Q45_6 Q45_7 Q45_8 Q54 Q55 Q56_Q56_1 Q56_Q56_2 Q56_Q56_3 Q56_Q56_4 Q57 Q58_Q58_1 Q58_Q58_2 Q58_Q58_3 Q58_Q58_4 Q63 Q64 Q67 Q74 Q75 Q76 Q77 Q85_Q85_1 Q85_Q85_2 Q85_Q85_3 Q85_Q85_4 Q86_Q86_1 Q86_Q86_2 Q86_Q86_3 Q86_Q86_4 Q89 Q90 Q94 Q95 Q96 Q97 Q98 Q99 Q100 Q102 Q104 Q106 Q107_1 Q107_2 Q107_3 Q107_4 Q107_5 Q108 Q109 Q110 Q1100

*Dropped Nepal*

StartDate EndDate Status IPAddress Progress Duration__in_seconds_ Finished RecordedDate ResponseId RecipientLastName RecipientFirstName RecipientEmail ExternalReference LocationLatitude LocationLongitude DistributionChannel UserLanguage Q1 Q30 Q32 Q34 Q43 Q44 Q45 Q46 Q47 Q48 Q50 Q52 Q53 Q57 Q58_1 Q58_2 Q58_3 Q58_4 Q58_88 Q63_1 Q63_2 Q63_3 Q63_4 Q63_5 Q63_6 Q63_7 Q63_8 Q64 Q65_1 Q65_2 Q65_3 Q65_4 Q65_5 Q65_6 Q65_7 Q65_8 Q65_9 Q66_1 Q66_2 Q66_3 Q66_4 Q66_5 Q67_1 Q67_2 Q67_3 Q67_4 Q67_4 Q67_5 Q67_6 Q67_7 Q67_8 Q69 Q70_1 Q70_2 Q70_3 Q70_4 Q70_5 Q70_6 Q70_7 Q70_8 Q71 Q72_1 Q72_2 Q72_3 Q72_4 Q72_5 Q72_6 Q72_7 Q72_8 Q81 Q82 Q83_1 Q83_2 Q83_3 Q83_4 Q84 Q85_1 Q85_2 Q85_3 Q85_4 Q90 Q91 Q94 Q97 Q102 Q103 Q105 Q106 Q111 Q112_1 Q112_2 Q112_3 Q112_4 Q112_5 Q112_6 Q112_7 Q112_8 Q112_9 Q112_10 Q112_88 Q120_1 Q120_2 Q120_3 Q120_4 Q120_5 Q120_6 Q120_7 Q120_8 Q120_9 Q120_10 Q120_11 Q121 Q122 Q123 Q124_1 Q124_2 Q124_3 Q124_4 Q124_5 Q124_6 Q124_7 Q124_8 Q125 Q127 Q128 Q129 Q130 Q131 Q132 Q133 Q135 Q137 Q139 Q140_1 Q140_2 Q140_3 Q140_4 Q140_5 Q141 Q142 Q143 SC0
