# Supplementary material for: Women, peace and insecurity: The risks of peacebuilding in everyday life for women in Sri Lanka and Nepal
Source: PLoS One. 2024 May 29;19(5):e0303023. doi: 10.1371/journal.pone.0303023 (PMC11135728; doi:10.1371/journal.pone.0303023)
Supplement: S1 Questionnaire — (PDF) [file pone.0303023.s002.pdf]

Sri Lanka Gender and Peacebuilding 2017

Survey Flow

Standard: Pre-survey ID observations by enumerator (Do not read) (6 Questions)
Standard: Consent form (6 Questions)
Standard: PDA explanation (2 Questions)
Block: Demographics (17 Questions)
Standard: Trauma stressors (14 Questions)
Standard: PTSD Symptoms (7 Questions)
Standard: Experiences of Family Violence (6 Questions)
Standard: Resilience factors (6 Questions)
Standard: Peacebuilding attitudes (29 Questions)
Standard: Gender Equality Attitudes (8 Questions)
Standard: End Survey (1 Question)
Standard: Validation (2 Questions)

Branch: New Branch
If
If Enter password Text Response Is Equal to 1234

Standard: Post-Survey Observation (7 Questions)

Page Break

Start of Block: Pre-survey ID observations by enumerator (Do not read)

Q1 Enumerator ID

____

Q2 District

▼ Vavuniya (1) ... Mannar (3)

Q3 DS Number

- Vengalacheddikulam (01)
- Vavuniya (02)
- Vavuniya South (03)
- Vavuniya North (04)
- Horowpothana (05)
- Mahavilachchiya (06)
- Padaviya (07)
- Medawachchiya (08)
- Kebithigollewa (09)
- Musalai (10)
- Nanaddan (11)
- Madhu (12)
- Manthai West (13)
- Mannar Town (14)

Q4 GN Number

▼ Chettikulam (1) ... Panankaddukoddu West (51)

Q5 Respondent's gender

○ Male (1)

○ Female (2)

Q6 Household number

▼ 1 (1) ... 99 (99)

End of Block: Pre-survey ID observations by enumerator (Do not read)

Start of Block: Consent form

Q7 I am [NAME] from [PLACE]. I am here to ask if you would be willing to participate in a research study directed and commissioned by a group of researchers in Sweden. The project leader is Dr. Karen Brounéus, who is an Associate Professor at Uppsala University in Sweden. You can reach her at the following address [GIVE CARD]. The research is conducted in collaboration with the University of Colombo.
This project aims at learning how people think and feel about issues such as the war, trust, coexistence, peace, and security. The end result of this project is to better understand the benefits and challenges of peacebuilding after war. The questionnaire is anonymous; the information will only be used for statistical purposes – no one will be able to identify you or your answers. The study is part of an academic research project, and it serves no other purpose; it is not affiliated with any government or political party. Your input would be highly valued and is greatly appreciated. You will enter your answers into an electronic device, but the responses you enter into the device cannot be traced to you.
The questionnaire will take around 45-60 minutes to complete. Your participation is completely voluntary; you are free to withdraw at any time. I hope you will participate, but choosing not to do so will not disadvantage you in any way.

Q8 May we have your permission to ask these questions, would you be willing to participate in this survey?

- Yes (1)
- No (2)

Skip To: Q10 If May we have your permission to ask these questions, would you be willing to participate in this s... = Yes

Display This Question:

If May we have your permission to ask these questions, would you be willing to participate in this s... = No

Q9 Thank you nevertheless for your time.

Skip To: End of Survey If Thank you nevertheless for your time.() Is Displayed

Q10 Thank you. What language would you like to take the survey in?

- Tamil (1)
- Sinhala (2)
- English (3)

Q11 Before we start, let me tell you a little bit about the process, since most people have not done a survey like this before. All of the answers will be entered on this tablet [SHOW RESPONDENT]. It is okay if you have not used a tablet like this before, I will work with you in the beginning and show you how to use it until you feel comfortable doing it yourself. Some of the questions will be about personal or sensitive issues. For these questions, it is best if you can try to enter the answers yourself so they remain private. However, I will be here to help you if you need it. If you fill out the survey by yourself on the tablet, you will have complete privacy. I can also help by reading the questions to you and you enter the answers yourself. Even if I help you, your answers will still be completely anonymous - I will not share that information with anyone else.

Q12
For each question, you will be given a set of answers, and you will be asked to choose the one that is closest to your own view. Even though none of the answers may fit your ideas exactly, please choose the response closest to your view. Some of the questions may seem similar, but please think carefully about each of them. Take your time. It is important that you answer as accurately as you can. If you have any questions – about how to understand a question or how the tablet works, you can always ask me.

Thank you, then we will begin.

End of Block: Consent form

Start of Block: PDA explanation

Q13

Let me briefly explain how the device works. First you will read the question, then select the most accurate response by tapping the response box or the circle next to it. To move to the next question, please press the arrow to the right. If you want to return to a previous question, please press the arrow to the left. Note that you sometimes need to scroll down the screen to see all of the information.

For some questions, you can choose many of the answers in the list – as many as apply to you. These questions will be in red letters, to remind you that you can select multiple options. These answers will also have a small box next to them instead of a circle.

Q16 Thank you. Now we will begin with the questions. I will work with you to answer the first set of questions. Once you have seen how the device works during this section, I will give you the device to enter the answers on your own. Throughout the survey, I will be here to help you with any questions.

End of Block: PDA explanation

Start of Block: Demographics

Q17 We would like to start by asking you a few questions about yourself.

Q18 Can you tell me your age, please?

- 20 or below (1)
- 21-30 (2)
- 31-40 (3)
- 41-50 (4)
- 51-60 (5)
- 61 or above (6)
- Do not know (88)

Q19 What is the highest education level you have achieved?

- No formal school (1)
- Between grades 1-5 (2)
- Between grades 6-11 (3)
- Up to Advanced level (4)
- Bachelor's Degree (5)
- Master’s degree or professional degree (6)
- Do not know (88)

Q20 What is your marital status?

- Single (1)
- Married (2)
- Deserted by spouse/partner (3)
- Separated (4)
- Divorced (5)
- Widowed (6)
- Spouse/partner missing (7)
- Do not know (88)

Display This Question:

If What is your marital status? = Widowed

X→

Q21 Did you lose your spouse/partner due to:

- The war (1983-2009) (1)
- Tsunami (2)
- Illness (3)
- Accident (4)
- Disappearance (5)
- Political Violence other than the war between LTTE & Government (6)
- Old age (7)
- Other (8)
- Do not know (88)

Q22 Do you have any children?

- Yes (1)
- No (2)
- Do not know (88)

Q23 What is your religion?

- ◯ Buddhist (1)
- ◯ Hindu (2)
- ◯ Muslim (3)
- ◯ Roman Catholic (4)
- ◯ Christian (Non-Roman Catholic) (5)
- ◯ No religion (6)
- ◯ Other (7)
- ◯ Do not know (88)

Q24 What is your ethnicity?

- ◯ Sinhala (1)
- ◯ Tamil (2)
- ◯ Muslim (3)
- ◯ Up-Country Tamil (4)
- ◯ Other (5)

Display This Question:

If Thank you. What language would you like to take the survey in? = Sinhala

Or Thank you. What language would you like to take the survey in? = English

Q25 Do you speak Tamil?

◯ Yes (1)

◯ No (2)

Display This Question:

If Thank you. What language would you like to take the survey in? = Tamil

Or Thank you. What language would you like to take the survey in? = English

X→

Q26 Do you speak Sinhala?

◯ Yes (1)

◯ No (2)

X→

Q27 What is your current employment status?

- ○ Self-employed (1)
- ○ Full or part time employed (2)
- ○ Retired (3)
- ○ Student (4)
- ○ Unemployed (5)
- ○ Performing home duties full time (6)
- ○ Working in agriculture (7)
- ○ Not working for other reasons like illness, disability, etc (8)
- ○ Other (9)
- ○ Do not know (88)

Q28 Thinking about your general physical health [things like: sickness, illness, injury, disease etc.] – on a scale from 1, poor, to 4, very good, how would you describe your overall physical health today?

- ○ 1. Very Poor (1)
- ○ 2. Somewhat Poor (2)
- ○ 3. Somewhat Good (3)
- ○ 4. Very Good (4)

Display This Question:

If Thinking about your general physical health [things like: sickness, illness, injury, disease etc.... = 1. Very Poor

Or Thinking about your general physical health [things like: sickness, illness, injury, disease etc.... = 2. Somewhat Poor

Q29 Approximately how long have you felt this way?

- A few days (1)
- A few weeks (2)
- A few months (3)
- A few years (4)
- For a very long time (5)
- Do not know (88)

Q30 Thinking about your general mental health [things like: anxiety, depression, fear, fatigue, tiredness, hopelessness etc.] – on a scale from 1, poor, to 4, very good, how would you describe your overall mental health today?

- 1. Very Poor (1)
- 2. Somewhat Poor (2)
- 3. Somewhat Good (3)
- 4. Good (4)

Display This Question:

If Thinking about your general mental health [things like: anxiety, depression, fear, fatigue, tired... = 1. Very Poor

Or Thinking about your general mental health [things like: anxiety, depression, fear, fatigue, tired... = 2. Somewhat Poor

Q31 Approximately how long have you felt this way?

- ○ A few days (1)
- ○ A few weeks (2)
- ○ A few months (3)
- ○ A few years (4)
- ○ For a very long time (5)
- ○ Do not know (88)

Q114 Thank you. Now we have gone through some questions together. I hope you feel comfortable using the tablet and selecting answers. Now, you can begin entering the answers on your own. Would you like me to leave you in privacy or would you like me to stay and read the questions aloud or provide other assistance?

- ○ Leave in privacy (1)
- ○ Stay and assist (2)

Display This Question:

If Thank you. Now we have gone through some questions together. I hope you feel comfortable using th... = Leave in privacy

Q115 I will now leave you in privacy and let you answer the survey questions. I will wait here until you have completed the survey. Please let me know if you require any assistance.

End of Block: Demographics

Q32 Thank you. Now, the following questions will ask about some personal experiences of conflict and violence, first during the period between 1983-2009 and second during this past year. Please mark all of the events you have experienced. Some of these questions may address sensitive issues. As always, your answers are anonymous.

Q33 Did you reside in Sri Lanka during the conflict in 1983-2009?

- ○ Yes (1)
- ○ No (2)

Q34 **Please think back to the period between 1983-2009, during which Sri Lanka experienced conflict between the Government of Sri Lanka and the LTTE. Did you experience any of the following during this period?**
(Select all that apply)

- ▢ Displacement (1)
- ▢ Belongings stolen (2)
- ▢ Belongings destroyed (3)
- ▢ House destroyed (4)
- ▢ House/land seized (5)
- ▢ I have not experienced any of the above (6)

Q35 **Still thinking of the period between 1983-2009, did you experience any of the following during this period?**
(Select all that apply):

- ☐ Threatened with violence (1)
- ☐ Threatened with death (2)
- ☐ Witnessed violence (3)
- ☐ Beaten violently or tortured (4)
- ☐ Forced to commit violence (5)
- ☐ Experienced sexual violence or sexual abuse (6)
- ☐ Witnessed sexual violence or sexual abuse (7)
- ☐ Forced to commit sexual violence or sexual abuse (8)
- ☐ I have not experienced any of the above (9)

Q36 **Again, still thinking of the period between 1983-2009, did any of your family members (spouse/child/parent) experience any of the following?**
(Select all that apply)

- ☐ Family member (Spouse/Child/Parent) injured (1)
- ☐ Family member (Spouse/Child/Parent) killed (2)
- ☐ Family member (Spouse/Child/Parent) abducted (3)
- ☐ Family member (Spouse/Child/Parent) disabled (4)
- ☐ I have not experienced any of the above (5)

Q37 **Still thinking of the period between 1983-2009, did you experience any of the following?**
(Select all that apply)

- ▢ Saw your loved ones dying in front of you (1)
- ▢ Imprisoned (2)
- ▢ Injured by land mines (3)
- ▢ Wounded due to shell attacks/ bomb blasts/ unexpected attacks (4)
- ▢ I have not experienced any of the above (5)

Q38 **If you experienced any of the events mentioned in the past questions, why do you think these things happened to you?**
(Select all that apply)

- ▢ People were bad at that time (1)
- ▢ There was a lot of hatred (2)
- ▢ I broke a law (3)
- ▢ I did something bad (4)
- ▢ I was unlucky (5)
- ▢ Karma (6)
- ▢ Other (7)
- ▢ Not relevant (8)

Q39 How often do you think about your experiences during the war?

- ○ Never (1)
- ○ A few times a year (2)
- ○ A few times a month (3)
- ○ Every week (4)
- ○ Every day (5)
- ○ All of the time (6)

Q40 **Now I want you to think back over the past year. Did you experience any of the following during the past year [past twelve months]?**
(Select all that apply)

- ☐ Displacement (1)
- ☐ Belongings stolen (2)
- ☐ Belongings destroyed (3)
- ☐ House destroyed (4)
- ☐ Threatened with violence (5)
- ☐ Threatened with death (6)
- ☐ Imprisoned (7)
- ☐ Injured by land mines (8)
- ☐ I have not experienced any of the above (10)

Q41 **Still thinking about the past year [past twelve months], did you experience any of the following?**
(Select all that apply)

- ☐ Family member (Spouse/Child/Parent) injured (1)
- ☐ Family member (Spouse/Child/Parent) killed (2)
- ☐ Family member (Spouse/Child/Parent) abducted (3)
- ☐ I have not experienced any of the above (5)

Q42 **Again, thinking about the past year [past twelve months], did you experience any of the following?**
(Select all that apply)

- [ ] Saw your loved ones dying in front of you (1)
- [ ] Witnessed violence (2)
- [ ] Beaten violently or tortured (3)
- [ ] Forced to commit violence (4)
- [ ] Experienced sexual violence or sexual abuse (5)
- [ ] Witnessed sexual violence or sexual abuse (6)
- [ ] Forced to commit sexual violence or sexual abuse (7)
- [ ] I have not experienced any of the above (8)

Q43 Thank you for answering these questions, you are doing very well. The next question will ask about your current situation in terms of your personal safety and security.

What do you perceive to be the greatest threat you currently face to your safety?

- ◯ Renewed violence in my community (1)
- ◯ Domestic abuse or violence (2)
- ◯ Physical violence against myself, other than domestic violence (3)
- ◯ Struggle for economic wellbeing (food, shelter, healthcare) (4)
- ◯ Sexual abuse or violence (5)
- ◯ Abduction (6)
- ◯ Religious or ethnic tensions (7)
- ◯ Access to safe drinking water (8)
- ◯ Natural disasters (9)
- ◯ Human-elephant conflict (10)
- ◯ Other (11)
- ◯ I currently do not face any threats to my safety (12)

Q44 Were you affected in any way by a flood or drought in the last five years?

- ◯ Yes (1)
- ◯ No (2)
- ◯ Do not know (88)

Display This Question:

If Were you affected in any way by a flood or drought in the last five years? = Yes

Q45 **How were you affected?**
(Select all that apply)

- ▢ Displacement (1)
- ▢ Property destroyed (2)
- ▢ Death of family member/s (3)
- ▢ Injury (4)
- ▢ Mental stress (5)
- ▢ Famine/starvation (6)
- ▢ Poverty (7)
- ▢ Other (8)

End of Block: Trauma stressors

Start of Block: PTSD Symptoms

Q46
The following questions ask about different problems that people sometimes have. For each one we would like to know how much you have experienced each one IN THE LAST MONTH, including today.

Q47 Repeated, disturbing memories, thoughts, or images of a stressful experience from the past?

- ◯ Not at all (1)
- ◯ A little bit (2)
- ◯ Moderately (3)
- ◯ Quite a bit (4)
- ◯ Extremely (5)

Q48 Feeling very upset when something reminded you of a stressful experience from the past?

- ◯ Not at all (1)
- ◯ A little bit (2)
- ◯ Moderately (3)
- ◯ Quite a bit (4)
- ◯ Extremely (5)

Q49
Avoid activities or situations because they remind you of a stressful experience from the past?

- Not at all (1)
- A little bit (2)
- Moderately (3)
- Quite a bit (4)
- Extremely (5)

Q50
Feeling distant or cut off from other people?

- Not at all (1)
- A little bit (2)
- Moderately (3)
- Quite a bit (4)
- Extremely (5)

Q51
Feeling irritable or having angry outbursts?

○ Not at all (1)

○ A little bit (2)

○ Moderately (3)

○ Quite a bit (4)

○ Extremely (5)

Q52
Having difficulty concentrating?

○ Not at all (1)

○ A little bit (2)

○ Moderately (3)

○ Quite a bit (4)

○ Extremely (5)

End of Block: PTSD Symptoms

Start of Block: Experiences of Family Violence

Q53 Now we would like to ask a few more questions about the types of violence that some people may experience at home. These questions will ask about three different types of violence: Physical, Emotional and Sexual.

Physical violence is when someone hits, slaps, kicks, punches or anything else to hurt you physically.

Emotional violence is when someone says or does something to humiliate you in front of others, threatens to harm you, or insults you and makes you feel bad about yourself.

Sexual violence is when someone forces you in any way to have sexual intercourse or perform any other sexual acts.

As always, your answers to these questions will be kept secret and will not be shared with anyone else.

Q54 Have you ever been subject to violence (Physical, Emotional and Sexual) at home?

- ◯ Yes (1)
- ◯ No (2)

Skip To: Q57 If Have you ever been subject to violence (Physical, Emotional and Sexual) at home? = No

---

Display This Question:

If Have you ever been subject to violence (Physical, Emotional and Sexual) at home? = Yes

Q55 Did this happen to you as an adult and/or as a child?

- ◯ Child (before age 18) (1)
- ◯ Adult (after age 18) (2)
- ◯ Both (before and after age 18) (3)

Display This Question:

If Have you ever been subject to violence (Physical, Emotional and Sexual) at home? = Yes

X→

Q56 **What type of violence? (Select all that apply)**

	Yes (1)	No (2)
Physical (hitting, slapping, kicking, or anything else to hurt you physically) (Q56_1)	○	○
Emotional (humiliation, threats of harm, insults) (Q56_2)	○	○
Sexual (forcing in any way to have sexual intercourse or perform any other sexual acts) (Q56_3)	○	○
Other (Q56_4)	○	○

X→

Q57 Have you ever subjected one of your family members to violence (Physical, Emotional, or Sexual) at home?

○ Yes (1)

○ No (2)

Skip To: End of Block If Have you ever subjected one of your family members to violence (Physical, Emotional, or Sexual) a... = No

---

Display This Question:

If Have you ever subjected one of your family members to violence (Physical, Emotional, or Sexual) a... = Yes

X→

Q58 **What type of violence?**
(Select all that apply)

	Yes (1)	No (2)
Physical (hitting, slapping, kicking, or anything else to cause physical hurt) (Q58_1)	○	○
Emotional (humiliation, threats of harm, insults) (Q58_2)	○	○
Sexual (forcing in any way to have sexual intercourse or perform any other sexual acts) (Q58_3)	○	○
Other (Q58_4)	○	○

End of Block: Experiences of Family Violence

Start of Block: Resilience factors

Q59 We know some of those were difficult questions to answer. Thank you for doing so, your answers are really important. The following questions will ask about your outlook on life, your social support networks, and your economic situation.

X→

Q60
How often do you have someone to confide in or talk to about yourself or your problems?

- Never (1)
- Rarely (2)
- Some of the time (3)
- Most of the time (4)
- All of the time (5)
- Do not know (88)

Q61 **When I feel lonely, abandoned, sad, deeply upset, feel down, angry, frustrated for different reasons I seek support from:**
(Select all that apply)

- Family Members, Elders, Village elders (1)
- Peers with similar experience (2)
- Religious Places/Leaders (3)
- Professionals (Doctors, Counsellors, Lawyers Social Workers) (4)
- Government institutions (Such as GN, Police, officers attached to the DS division) (5)
- Other (6)
- I do not have any place to go (7)
- I do not seek support at all (8)

Q62 How do you feel about your family’s current financial situation?

- Very good (1)
- Good (2)
- A little hard (3)
- Very hard (4)
- Do not know (88)

Q63 How economically independent are you?

- Very Much (1)
- Somewhat (2)
- Not at all (3)
- Do not know (88)

Q64 How is your economic situation compared with most of your neighbors'?

- ○ Much better (1)
- ○ Somewhat better (2)
- ○ Similar (3)
- ○ Somewhat worse (4)
- ○ Much worse (5)
- ○ Do not know (88)

End of Block: Resilience factors

Start of Block: Peacebuilding attitudes

Q65 You are progressing very well in the survey, thank you. The next questions will ask about your attitudes towards different issues related to peace.

Q66 A truth-telling process aims to uncover the facts and context of what happened during past conflict or violence. A truth-telling mechanism has been mentioned as a possible peacebuilding approach in Sri Lanka. Although other commissions and mechanisms have begun to address the conflict, a full truth-telling process has not happened yet. Thinking about future possibilities of truth-telling in Sri Lanka, please answer the following questions.

X→

Q67
For each statement, indicate how much you agree or disagree:

It is better not to open up old wounds by talking about what happened in the past.

- Strongly agree (1)
- Somewhat agree (2)
- Somewhat disagree (3)
- Strongly disagree (4)
- Do not know (88)

Q68 It is important to know what happened during the conflict and collect testimonies.

- Strongly agree (1)
- Somewhat agree (2)
- Somewhat disagree (3)
- Strongly disagree (4)
- Do not know (88)

Q69 Sharing the truth about conflict experiences would help to build more positive relationships between my ethnic group and *different* ethnic groups.

- Strongly agree (1)
- Somewhat agree (2)
- Somewhat disagree (3)
- Strongly disagree (4)
- Do not know (88)

Q70 The next question will ask about your own experience with different commissions and mechanisms that have happened in Sri Lanka, either in the past or that are now ongoing. Please answer from your own personal experience.

Q71 Have you given testimony to any of the following commissions or mechanisms? (Select all that apply)

- Commission of Inquiry (1)
- LLRC (2)
- Consultations before the Task Force on Reconciliation Mechanisms (3)
- Commission on Disappearances (4)
- Other (5)
- None of the above (6)

Skip To: Q73 If Have you given testimony to any of the following commissions or mechanisms? (Select all that apply) = None of the above

---

Q72 How satisfied are you with your experience of participating in this/these institution(s)?

- Extremely satisfied (1)
- Satisfied (2)
- Dissatisfied (3)
- Extremely dissatisfied (4)
- Mixed experiences (5)
- Do not know (88)

Q73 The next questions will ask your opinion on some common statements about yourself and society. For each statement, indicate how much you agree or disagree:

Q74 Generally, minorities are discriminated against, in their daily life.

- Strongly agree (1)
- Somewhat agree (2)
- Somewhat disagree (3)
- Strongly disagree (4)
- Do not know (88)

Q75 It is difficult for me to relate to members of other ethnic groups

- Strongly agree (1)
- Somewhat agree (2)
- Somewhat disagree (3)
- Strongly disagree (4)
- Do not know (88)

Q76 If I think I am right about something, it is of little consequence to me what other people think

- Strongly agree (1)
- Somewhat agree (2)
- Somewhat disagree (3)
- Strongly disagree (4)
- Do not know (88)

Q77 When I criticize someone, I think about how I would feel if I were in his/her place

- Strongly agree (1)
- Somewhat agree (2)
- Somewhat disagree (3)
- Strongly disagree (4)
- Do not know (88)

Q78
Next, we will ask some questions about trust.

Generally speaking, would you say that most people can be trusted or that you need to be very careful in dealing with people?

- Most people can be trusted (1)
- Need to be very careful (2)
- Do not know (88)

Q79 Do you think one should always be on guard towards other people, even if they are neighbours or friends?

- Yes (1)
- No (2)
- Do not know (88)

Q80 Do you think most people would try to take advantage of you if they got the chance, or would they try to be fair?

- ○ Most people would try to take advantage of you (1)
- ○ Most people would try to be fair (2)
- ○ Do not know (88)

Q81 We will ask a few more questions regarding trust. Remember that everything you share here will only be used for research purposes and will be kept strictly secret.

Q82 I'd like to ask you how much you trust people from various groups. Could you tell me for each whether you trust people from...

	Very much (1)	A little (2)	Not at all (3)	Do not know (88)
Your Family (Q82_1)	○	○	○	○
People in your neighborhood (Q82_2)	○	○	○	○
Sinhalese people (Q82_3)	○	○	○	○
Tamil people (Q82_4)	○	○	○	○
Muslim people (Q82_5)	○	○	○	○
People from another country (Q82_6)	○	○	○	○

Q83 To what extent do you feel threatened when you are around people from the following groups?

	Very much (1)	A little (2)	Not at all (3)	Do not know (88)
Sinhala (Q83_1)	○	○	○	○
Sri Lankan Tamil (Q83_2)	○	○	○	○
Muslim (Q83_3)	○	○	○	○
Up-Country Tamil (Q83_4)	○	○	○	○
Buddhist (Q83_5)	○	○	○	○
Hindu (Q83_6)	○	○	○	○
Roman Catholic (Q83_7)	○	○	○	○
Christian (Non-RC) (Q83_8)	○	○	○	○

Q84 Now, we will turn to some other questions. These questions may sound a bit similar, but we ask that you consider each option carefully.

For each statement, please indicate how much you agree or disagree.

Q85 To what extent do you feel that increasing numbers of each of these groups to your area will increase criminal rates?

	Very much (1)	A little (2)	Not at all (3)	Do not know (4)
Sinhala (Q85_1)	○	○	○	○
Sri Lankan Tamil (Q85_2)	○	○	○	○
Muslim (Q85_3)	○	○	○	○
Up-Country Tamil (Q85_4)	○	○	○	○

Q86 To what extent do you feel that sharing the same space and interacting with members of each of these groups will lead to the disappearance of the cultural identity of your own ethnic group?

	Very much (1)	A little (2)	Not at all (3)	Do not know (4)
Sinhala (Q86_1)	○	○	○	○
Sri Lankan Tamil (Q86_2)	○	○	○	○
Muslim (Q86_3)	○	○	○	○
Up-Country Tamil (Q86_4)	○	○	○	○

Q87 How comfortable would you feel in the following situations in the presence of current Sri Lanka Army soldiers?

	Completely comfortable (1)	Somewhat Comfortable (2)	Somewhat Uncomfortable (3)	Completely Uncomfortable (4)
Working with them (Q87_1)	◯	◯	◯	◯
Living in the same village/ community (Q87_2)	◯	◯	◯	◯
Living as close neighbors (Q87_3)	◯	◯	◯	◯
Marrying a family member (Q87_4)	◯	◯	◯	◯

Q88 How comfortable would you feel in the following situations in the presence of ex-LTTE cadres?

	Completely comfortable (1)	Somewhat Comfortable (2)	Somewhat Uncomfortable (3)	Completely Uncomfortable (4)
Working with them (Q88_1)	◯	◯	◯	◯
Living in the same village/ community (Q88_2)	◯	◯	◯	◯
Living as close neighbors (Q88_3)	◯	◯	◯	◯
Marrying a family member (Q88_4)	◯	◯	◯	◯

Q89 To what extent do you feel other people respect your own ethnic group?

- Very much respected (1)
- Somewhat respected (2)
- Somewhat disrespected (3)
- Very much disrespected (4)
- Do not know (88)

Q90 Now, please tell me how much you agree with the following statement: My ethnic group is discriminated against by others

- Strongly agree (1)
- Somewhat agree (2)
- Somewhat disagree (3)
- Strongly disagree (4)
- Do not know (88)

Q91
We would now like to ask about your views on responsibility. Please indicate how much you agree or disagree with the following statements.

All those who perpetrated violence in the 1983-2009 conflict, regardless of side, should be held responsible.

- Completely agree (1)
- Somewhat agree (2)
- Somewhat disagree (3)
- Completely disagree (4)
- Do not know (88)

Q92 **Should any of the following things happen to Sri Lanka Army soldiers who perpetrated violence in the 1983-2009 conflict?**

(Select all that apply)

- ▢ Nothing should happen to them (1)
- ▢ Punish them (2)
- ▢ Put them in jail (3)
- ▢ See them in trials / court (4)
- ▢ Want to see them dead (5)
- ▢ Have them compensate victims (6)
- ▢ They should ask for forgiveness (7)
- ▢ They should confess their crimes (8)
- ▢ They should be forgiven (9)
- ▢ Reintegrate them in the community (10)
- ▢ Other (11)

Q113 Should any of the following things happen to LTTE soldiers who perpetrated violence in the 1983-2009 conflict?

(Select all that apply)

- ▢ Nothing should happen to them (1)
- ▢ Punish them (2)
- ▢ Put them in jail (3)
- ▢ See them in trials / court (4)
- ▢ Want to see them dead (5)
- ▢ Have them compensate victims (6)
- ▢ They should ask for forgiveness (7)
- ▢ They should confess their crimes (8)
- ▢ They should be forgiven (9)
- ▢ Reintegrate them in the community (10)
- ▢ Other (11)

End of Block: Peacebuilding attitudes

Start of Block: Gender Equality Attitudes

Q93 The next questions are about the roles of men and women. This is a different topic than our previous questions, but this topic is also important to our study.

Q94 A man shouldn’t show emotions and weakness.

- Completely agree (1)
- Somewhat agree (2)
- Somewhat disagree (3)
- Completely disagree (4)
- Do not know (88)

Q95 It is manly to defend the honor of your family even by violent means.

- Completely agree (1)
- Somewhat agree (2)
- Somewhat disagree (3)
- Completely disagree (4)
- Do not know (88)

Q96 The male family members should make the final decision on all matters.

- Completely agree (1)
- Somewhat agree (2)
- Somewhat disagree (3)
- Completely disagree (4)
- Do not know (88)

Q97 There are times when a woman deserves to be beaten.

- Completely agree (1)
- Somewhat agree (2)
- Somewhat disagree (3)
- Completely disagree (4)
- Do not know (88)

Q98 Men are better suited to own land than women are.

- Completely agree (1)
- Somewhat agree (2)
- Somewhat disagree (3)
- Completely disagree (4)
- Do not know (88)

Q99 When jobs are scarce, men should have more right to a job than women.

- ○ Completely agree (1)
- ○ Somewhat agree (2)
- ○ Somewhat disagree (3)
- ○ Completely disagree (4)
- ○ Do not know (88)

Q100 If a woman earns more money than her husband, it's almost certain to cause problems.

- ○ Completely agree (1)
- ○ Somewhat agree (2)
- ○ Somewhat disagree (3)
- ○ Completely disagree (4)
- ○ Do not know (88)

End of Block: Gender Equality Attitudes

Start of Block: End Survey

Q101
Thank you, the survey is now finished! We greatly appreciate your participation. Please hand the device over to the interviewer.

End of Block: End Survey

Start of Block: Validation

Q102 Enter password

____

Display This Question:

If If Enter password Text Response Is Not Equal to 1234

Q103 I'm sorry, that ID number was incorrect. Please use the back button to return to the last question and re-enter the correct ID number.

End of Block: Validation

Start of Block: Post-Survey Observation

X→

Q104 Interview privacy; the respondent was left in privacy with no interruptions while tapping in answers.

○ Yes (1)

○ No (2)

Q105 PDA Use

○ Self-administered (1)

○ Partially self-administered, some assistance (2)

○ Enumerator assisted respondent throughout the survey (3)

X→

Q106 Did the respondent ask for assistance mid-interview?

○ Yes (1)

○ No (2)

Skip To: Q108 If Did the respondent ask for assistance mid-interview? = No

Display This Question:

If Did the respondent ask for assistance mid-interview? = Yes

Q107 What type of assistance? (Select all that apply)

- ▢ Technical assistance (related to the tablet) (1)
- ▢ Substantial understanding of the survey questions (2)
- ▢ Financial assistance (3)
- ▢ Other material assistance (4)
- ▢ Other (5)

Q108 As far as you can tell, did the respondent have an emotional reaction to the questions?

- ○ Yes (1)
- ○ No (2)

Skip To: Q110 If As far as you can tell, did the respondent have an emotional reaction to the questions? = No

Display This Question:

If As far as you can tell, did the respondent have an emotional reaction to the questions? = Yes

Q109 Please specify

____

Q110 Is there anything else to note?

____

End of Block: Post-Survey Observation
