# Supplementary material for: Women, peace and insecurity: The risks of peacebuilding in everyday life for women in Sri Lanka and Nepal
Source: PLoS One. 2024 May 29;19(5):e0303023. doi: 10.1371/journal.pone.0303023 (PMC11135728; doi:10.1371/journal.pone.0303023)
Supplement: S2 Questionnaire — (PDF) [file pone.0303023.s003.pdf]

Nepal Gender and Peacebuilding 2018

Survey Flow

Standard: Pre-survey ID observations by enumerator (Do not read) (30 Questions)
Standard: Consent form (6 Questions)
Standard: PDA explanation (2 Questions)
Block: Demographics (16 Questions)
Standard: Trauma stressors (18 Questions)
Standard: PTSD Symptoms (7 Questions)
Standard: Experiences of Family Violence (6 Questions)
Standard: Resilience factors (6 Questions)
Standard: Peacebuilding attitudes (34 Questions)
Standard: Gender Equality Attitudes (8 Questions)
Standard: End Survey (1 Question)
Standard: Validation (2 Questions)

Branch: New Branch
  If
    If Enter password Text Response Is Equal to 1234

  Standard: Post-Survey Observation (7 Questions)

Page Break

Start of Block: Pre-survey ID observations by enumerator (Do not read)

Q1 Enumerator ID

____

Q2 District

- ◯ Jhapa (1)
- ◯ Morang (2)
- ◯ Sunsari (3)
- ◯ Dang (4)
- ◯ Bardiya (5)
- ◯ Surkhet (6)

Display This Question:

If District = Jhapa

Q3 Jhapa - select municipality/VDC

- ◯ Arjundhara (1)
- ◯ Bahundangi (2)
- ◯ Gauriganj (3)

Display This Question:

If District = Morang

Q4 Morang - select municipality/VDC

- ○ Amardaha (1)
- ○ Madhumalla (2)
- ○ Pathari (3)

Display This Question:

If District = Sunsari

Q5 Sunsari- select municipality/VDC

- ○ Aurabani (1)
- ○ Madhelee (2)

Display This Question:

If District = Dang

Q6 Dang - select municipality/VDC

- ○ Chaulahi (1)
- ○ Lalmatiya (2)
- ○ Rampur (3)
- ○ Ghorahi Municipality (4)

Display This Question:

If District = Bardiya

Q7 Bardiya - select municipality/VDC

- Dhadhawar (1)
- Gola (2)
- Motipur (3)
- Magargadhi (4)

Display This Question:

If District = Surkhet

Q8 Surkhet- select municipality/VDC

- Birendranagar Municipality (1)
- Gumi (2)
- Latikoili (3)
- Chhinchu (4)

Display This Question:

If Jhapa - select municipality/VDC = Arjundhara

Q9 Arjundhara - select ward

- ◯ 2 (2)
- ◯ 4 (4)
- ◯ 5 (5)
- ◯ 7 (7)
- ◯ 9 (9)

Display This Question:

If Jhapa - select municipality/VDC = Bahundangi

Q10 Bahundangi- select ward

- ◯ 2 (2)
- ◯ 3 (3)
- ◯ 5 (5)
- ◯ 7 (7)
- ◯ 9 (9)

Display This Question:

If Jhapa - select municipality/VDC = Gauriganj

Q11 Gauriganj - select ward

- ○ 2 (2)
- ○ 4 (4)
- ○ 5 (5)
- ○ 7 (7)
- ○ 8 (8)

Display This Question:

If Morang - select municipality/VDC = Amardaha

Q12 Amardaha - select ward

- ○ 2 (2)
- ○ 5 (5)
- ○ 6 (6)
- ○ 7 (7)
- ○ 9 (9)

Display This Question:

If Morang - select municipality/VDC = Madhumalla

Q13 Madhumalla - select ward

- 1 (1)
- 2 (2)
- 4 (4)
- 7 (7)
- 9 (9)

Display This Question:

If Morang - select municipality/VDC = Pathari

Q14 Pathari - select ward

- 1 (1)
- 2 (2)
- 4 (4)
- 7 (7)
- 9 (9)

Display This Question:

If Sunsari- select municipality/VDC = Aurabani

Q15 Aurabani - select ward

- 1 (1)
- 3 (3)
- 5 (5)
- 7 (7)
- 9 (9)

Display This Question:

If Sunsari- select municipality/VDC = Madhelee

Q16 Madhelee - select ward

- 2 (2)
- 3 (3)
- 5 (5)
- 7 (7)
- 8 (8)

Display This Question:

If Dang - select municipality/VDC = Chaulahi

Q17 Chaulahi - select ward

- ◯ 2 (2)
- ◯ 3 (3)
- ◯ 7 (7)
- ◯ 9 (9)

Display This Question:

If Dang - select municipality/VDC = Lalmatiya

Q18 Lalmatiya - select ward

- ◯ 1 (1)
- ◯ 3 (3)
- ◯ 4 (4)
- ◯ 7 (7)
- ◯ 8 (8)

Display This Question:

If Dang - select municipality/VDC = Rampur

Q19 Rampur - select ward

○ 1 (1)

○ 3 (3)

○ 5 (5)

○ 7 (7)

○ 9 (9)

Display This Question:

If Dang - select municipality/VDC = Ghorahi Municipality

Q20 Ghorahi Municipality - select ward

○ 2 (2)

○ 6 (6)

○ 9 (9)

○ 10 (10)

○ 11 (11)

Display This Question:

If Bardiya - select municipality/VDC = Dhadhawar

Q21 Dhadhawar - select ward

- 2 (2)
- 4 (4)
- 6 (6)
- 8 (8)
- 9 (9)

Display This Question:

If Bardiya - select municipality/VDC = Gola

Q22 Gola - select ward

- 2 (2)
- 4 (4)
- 5 (5)
- 7 (7)
- 8 (8)

Display This Question:

If Bardiya - select municipality/VDC = Motipur

Q23 Motipur - select ward

- 1 (1)
- 4 (4)
- 6 (6)
- 7 (7)
- 8 (8)

Display This Question:

If Bardiya - select municipality/VDC = Magargadhi

Q24 Magargadhi - select ward

- 1 (1)
- 3 (3)
- 4 (4)
- 6 (6)
- 9 (9)

Display This Question:

If Surkhet- select municipality/VDC = Birendranagar Municipality

Q25 Birendranagar Municipality- select ward

◯ 1 (1)

◯ 3 (3)

◯ 5 (5)

◯ 6 (6)

◯ 8 (8)

Display This Question:

If Surkhet- select municipality/VDC = Gumi

Q26 Gumi - select ward

◯ 2 (2)

◯ 3 (3)

◯ 5 (5)

◯ 6 (6)

◯ 8 (8)

Display This Question:

If Surkhet- select municipality/VDC = Latikoili

Q27 Latikoili - select ward

- 1 (1)
- 3 (3)
- 7 (7)
- 8 (8)
- 9 (9)

Display This Question:

If Surkhet- select municipality/VDC = Chhinchu

Q28 Chhinchu - select ward

- 1 (1)
- 3 (3)
- 6 (6)
- 7 (7)
- 8 (8)

Q29 Respondent's gender

- Male (1)
- Female (2)

Q30 Household number

▼ 1 (1) ... 99 (99)

End of Block: Pre-survey ID observations by enumerator (Do not read)

Start of Block: Consent form

Q31 I am [NAME] from [PLACE]. I am here to ask if you would be willing to participate in a research study directed and commissioned by a group of researchers in Sweden and Nepal. The project leader is Dr. Karen Brounéus, who is an Associate Professor at Uppsala University in Sweden. You can reach her at the following address [GIVE CARD]. The research is conducted in collaboration with the Center for Social Change in Kathmandu. The project leader in Kathmandu is Dr. Prakash Bhattarai.

This project aims at learning how people think and feel about issues such as the war, trust, coexistence, peace, and security. The end result of this project is to better understand the benefits and challenges of peacebuilding after war. The questionnaire is anonymous; the information will only be used for statistical purposes – no one will be able to identify you or your answers. The study is part of an academic research project, and it serves no other purpose; it is not affiliated with any government or political party. Your input would be highly valued and is greatly appreciated. You will enter your answers into an electronic device, but the responses you enter into the device cannot be traced to you.

The questionnaire will take around 45-60 minutes to complete. Your participation is completely voluntary. If you choose to participate, you can skip a question if it makes you uncomfortable. You can also withdraw from the survey at any time. I hope you will participate, but choosing not to do so will not disadvantage you in any way.

Q32 May we have your permission to ask these questions, would you be willing to participate in this survey?

○ Yes (1)

○ No (2)

Skip To: Q34 If May we have your permission to ask these questions, would you be willing to participate in this s... = Yes

Display This Question:

If May we have your permission to ask these questions, would you be willing to participate in this s... = No

Q33 Thank you nevertheless for your time.

Skip To: End of Survey If Thank you nevertheless for your time.() Is Displayed

---

Q34 Thank you. What language would you like to take the survey in?

- ◯ Nepali (1)
- ◯ English (2)

Q35 Before we start, let me tell you a little bit about the process, since most people have not done a survey like this before. All of the answers will be entered on this tablet [SHOW RESPONDENT]. It is okay if you have not used a tablet like this before, I will work with you in the beginning and show you how to use it until you feel comfortable doing it yourself. Some of the questions will be about personal or sensitive issues. For these questions, it is best if you can try to enter the answers yourself so they remain private. However, I will be here to help you if you need it. If you fill out the survey by yourself on the tablet, you will have complete privacy. I can also help by reading the questions to you and you enter the answers yourself. Even if I help you, your answers will still be completely anonymous - I will not share that information with anyone else.

Q36
For each question, you will be given a set of answers, and you will be asked to choose the one that is closest to your own view. Even though none of the answers may fit your ideas exactly, please choose the response closest to your view. Some of the questions may seem similar, but please think carefully about each of them. Take your time. It is important that you answer as accurately as you can. If you have any questions – about how to understand a question or how the tablet works, you can always ask me.

Thank you, then we will begin.

End of Block: Consent form

Start of Block: PDA explanation

Q37

Let me briefly explain how the device works. First you will read the question, then select the most accurate response by tapping the response box. To move to the next question, please press the arrow to the right. If you want to return to a previous question, please press the arrow to the left. Note that you sometimes need to scroll down the screen to see all of the information.

For some questions, you can choose many of the answers in the list – as many as apply to you. These questions will be in red letters, to remind you that you can select multiple options.

Q38 Thank you. Now we will begin with the questions. I will work with you to answer the first set of questions. Once you have seen how the device works during this section, I will give you the device to enter the answers on your own. Throughout the survey, I will be here to help you with any questions.

End of Block: PDA explanation

Start of Block: Demographics

Q39 We would like to start by asking you a few questions about yourself.

Q40 Can you tell me your age, please?

- 20 or below (1)
- 21-30 (2)
- 31-40 (3)
- 41-50 (4)
- 51-60 (5)
- 61 or above (6)
- Do not know (88)

Q41 What is the highest education level you have achieved?

- No formal school (1)
- Primary school (2)
- Secondary school (3)
- High School (4)
- Bachelor's degree (5)
- Master’s degree or PhD (6)
- Professional degree (7)
- Do not know (88)

Q42 What is your marital status?

- ◯ Single (1)
- ◯ Married (2)
- ◯ Divorced (3)
- ◯ Widowed (4)
- ◯ Spouse/partner missing (5)
- ◯ Deserted by spouse/partner (6)
- ◯ Do not know (88)

Display This Question:

If What is your marital status? = Widowed

X→

Q43 Did you lose your spouse/partner due to:

- ◯ The armed conflict (1996-2006) (1)
- ◯ The earthquake in 2015 (2)
- ◯ Illness (3)
- ◯ Accident (4)
- ◯ Disappearance/missing (5)
- ◯ Political violence/protest other than the armed conflict between CPN-Maoist and the then government (6)
- ◯ Old age (7)
- ◯ Other (8)
- ◯ Do not know (88)

Q44 Do you have any children?

- ◯ Yes (1)
- ◯ No (2)
- ◯ Do not know (88)

Q45 What is your religion?

- ○ Hindu (1)
- ○ Buddhist (2)
- ○ Muslim (3)
- ○ Christian (4)
- ○ Kirat (5)
- ○ No religion (6)
- ○ Other (7)
- ○ Do not know (88)

Q46 Which caste or ethnic group do you belong to?

- Hill-Brahmin (1)
- Hill-Chhetri (2)
- Terai Brahmin/Chhetri (3)
- Other Terai caste (4)
- Hill Dalit (5)
- Terai Dalit (6)
- Newar (7)
- Indigenous (8)
- Hill Janajati (9)
- Terai Janajati (10)
- Muslim (11)
- Other (12)
- Do not know (88)

Q47 What language or languages do you speak most often at home?

- Nepali (1)
- Maithili (2)
- Bhojpuri (3)
- Tharu (4)
- Tamang (5)
- Newari (6)
- Magar (7)
- Urdu (8)
- Abadhi (9)
- Doteli (10)
- Two or more languages (11)
- Other (12)

Q48 What is your current employment status?

- ○ Self-employed (1)
- ○ Full time employed (2)
- ○ Part time employed (3)
- ○ Retired (4)
- ○ Student (5)
- ○ Unemployed (6)
- ○ Performing home duties full time (7)
- ○ Working in agriculture (8)
- ○ Not working for other reasons like illness, disability, etc (9)
- ○ Other (10)
- ○ Do not know (88)

X→

Q49 Thinking about your general physical health [things like: sickness, illness, injury, disease etc.] – on a scale from 1, very poor, to 4, very good, how would you describe your overall physical health today?

- ○ 1. Very Poor (1)
- ○ 2. Somewhat Poor (2)
- ○ 3. Somewhat Good (3)
- ○ 4. Very Good (4)

Display This Question:

If Thinking about your general physical health [things like: sickness, illness, injury, disease etc.... = 1. Very Poor

Or Thinking about your general physical health [things like: sickness, illness, injury, disease etc.... = 2. Somewhat Poor

Q50 Approximately how long have you felt this way?

- A few days (1)
- A few weeks (2)
- A few months (3)
- A few years (4)
- For a very long time (5)
- Do not know (88)

Q51 Thinking about your general mental health [things like: anxiety, depression, fear, fatigue, tiredness, hopelessness etc.] – on a scale from 1, very poor, to 4, very good, how would you describe your overall mental health today?

- 1. Very Poor (1)
- 2. Somewhat Poor (2)
- 3. Somewhat Good (3)
- 4. Very Good (4)

Display This Question:

If Thinking about your general mental health [things like: anxiety, depression, fear, fatigue, tired... = 1. Very Poor

Or Thinking about your general mental health [things like: anxiety, depression, fear, fatigue, tired... = 2. Somewhat Poor

Q52 Approximately how long have you felt this way?

- ○ A few days (1)
- ○ A few weeks (2)
- ○ A few months (3)
- ○ A few years (4)
- ○ For a very long time (5)
- ○ Do not know (88)

Q53 Thank you. Now we have gone through some questions together. I hope you feel comfortable using the tablet and selecting answers. Now, you can begin entering the answers on your own. Would you like me to leave you in privacy or would you like me to stay and read the questions aloud or provide other assistance?

- ○ Leave in privacy (1)
- ○ Stay and assist (2)

Display This Question:

If Thank you. Now we have gone through some questions together. I hope you feel comfortable using th... = Leave in privacy

Q54 I will now leave you in privacy and let you answer the survey questions. I will wait here until you have completed the survey. Please let me know if you require any assistance.

End of Block: Demographics

Start of Block: Trauma stressors

Q55 Thank you. Now, the following questions will ask about some personal experiences of conflict and violence, first during the period between 1996-2006 and second during this past year. Please mark all of the events you have experienced. Some of these questions may address sensitive issues. As always, your answers are anonymous.

X→

Q56 Did you reside in Nepal during the conflict in 1996-2006?

- Yes (1)
- No (2)

X→

Q57 Did you participate as a combatant in the conflict 1996-2006?

- Yes (1)
- No (2)
- Do not know (88)

Display This Question:

If Did you participate as a combatant in the conflict 1996-2006? = Yes

X→

Q58 **In which group(s) did you participate? Select all that apply.**

- ☐ Government security forces (1)
- ☐ The Maoist Army (2)
- ☐ Groups associated with a political party (other than the Maoists) (3)
- ☐ Other (4)
- ☐ Do not know (88)

Q59 **Please think back to the period between 1996-2006, during which Nepal experienced conflict between the Government of Nepal and the CPN-M. Which of the following experiences did you have?**
(Select all that apply)

- ☐ Displacement (1)
- ☐ Belongings stolen (2)
- ☐ Belongings destroyed (3)
- ☐ House destroyed (4)
- ☐ House/land seized (5)
- ☐ House raid (6)
- ☐ Forced donation (e.g. monthly levy) (7)
- ☐ I have not experienced any of the above (8)

Q60 **Still thinking of the period between** 1996-2006**, did you experience any of the following during this period?**
(Select all that apply):

- ▢ Threatened with violence (1)
- ▢ Threatened with death (2)
- ▢ Witnessed violence (3)
- ▢ Beaten violently or tortured (4)
- ▢ Forced to commit violence (5)
- ▢ Experienced sexual violence or sexual abuse (6)
- ▢ Witnessed sexual violence or sexual abuse (7)
- ▢ Forced to commit sexual violence or sexual abuse (8)
- ▢ I have not experienced any of the above (9)

Q61 **Again, still thinking of the period between** 1996-2006**, did any of your family members (spouse/child/parent) experience any of the following?**
(Select all that apply)

- ☐ Family member (Spouse/Child/Parent) injured (1)
- ☐ Family member (Spouse/Child/Parent) killed (2)
- ☐ Family member (Spouse/Child/Parent) abducted (3)
- ☐ Family member (Spouse/Child/Parent) disabled (4)
- ☐ I have not experienced any of the above (5)

Q62 **Still thinking of the period between** 1996-2006**, did you experience any of the following?**
(Select all that apply)

- ☐ Saw your loved ones dying in front of you (1)
- ☐ Imprisoned (2)
- ☐ Injured by land mines (3)
- ☐ Wounded due to shell attacks/ bomb blasts/ unexpected attacks (4)
- ☐ I have not experienced any of the above (5)

Q63 **If you experienced any of the events mentioned in the past questions, why do you think these things happened to you?**
(Select all that apply)

- ▢ People were bad at that time (1)
- ▢ There was a lot of hatred (2)
- ▢ I broke a law (3)
- ▢ I did something bad (4)
- ▢ I was unlucky (5)
- ▢ Karma (6)
- ▢ Other (7)
- ▢ Not relevant (8)

Q64 How often do you think about your experiences during the armed conflict period between 1996-2006?

- ○ Never (1)
- ○ A few times a year (2)
- ○ A few times a month (3)
- ○ Every week (4)
- ○ Every day (5)
- ○ All of the time (6)

Q65 **Now I want you to think back over the past year. Did you experience any of the following during the past year [past twelve months]?**
(Select all that apply)

- ☐ Displacement (1)
- ☐ Belongings stolen (2)
- ☐ Belongings destroyed (3)
- ☐ House destroyed (4)
- ☐ Threatened with violence (5)
- ☐ Threatened with death (6)
- ☐ Imprisoned (7)
- ☐ Injured by land mines (8)
- ☐ I have not experienced any of the above (9)

Q66 **Still thinking about the past year [past twelve months], did you experience any of the following?**
(Select all that apply)

- ☐ Family member (Spouse/Child/Parent) injured (1)
- ☐ Family member (Spouse/Child/Parent) killed (2)
- ☐ Family member (Spouse/Child/Parent) abducted (3)
- ☐ Family member (Spouse/Child/Parent) disabled (4)
- ☐ I have not experienced any of the above (5)

Q67 **Again, thinking about the past year [past twelve months], did you experience any of the following?**
(Select all that apply)

- ☐ Saw your loved ones dying in front of you (1)
- ☐ Witnessed violence (2)
- ☐ Beaten violently or tortured (3)
- ☐ Forced to commit violence (4)
- ☐ Experienced sexual violence or sexual abuse (5)
- ☐ Witnessed sexual violence or sexual abuse (6)
- ☐ Forced to commit sexual violence or sexual abuse (7)
- ☐ I have not experienced any of the above (8)

Q68 Thank you for answering these questions, you are doing very well. The next question will ask about your current situation in terms of your personal safety and security.

What do you perceive to be the greatest threat you currently face to your safety?

- ○ Renewed violence in my community (1)
- ○ Domestic abuse or violence (2)
- ○ Physical violence against myself, other than domestic violence (3)
- ○ Struggle for economic wellbeing (food, shelter, healthcare) (4)
- ○ Sexual abuse or violence (5)
- ○ Abduction (6)
- ○ Religious or ethnic tensions (7)
- ○ Scarcity of safe drinking water (8)
- ○ Natural disasters (9)
- ○ Threats from wild animals (10)
- ○ Other (11)
- ○ I currently do not face any threats to my safety (12)

Q69 Were you affected in any way by a flood or drought in the last five years?

- Yes (1)
- No (2)
- Do not know (88)

Display This Question:

If Were you affected in any way by a flood or drought in the last five years? = Yes

Q70 **How were you affected?**
(Select all that apply)

- Displacement (1)
- Property destroyed (2)
- Death of family member/s (3)
- Injury (4)
- Mental stress (5)
- Famine/starvation (6)
- Poverty (7)
- Other (8)

Q71 Were you in any way affected by the 2015 earthquake?

- Yes (1)
- No (2)
- Do not know (88)

Display This Question:

If Were you in any way affected by the 2015 earthquake? = Yes

Q72 **How were you affected? (Select all that apply)**

- Displacement (1)
- Property destroyed (2)
- Death of family member(s) (3)
- Injury (4)
- Mental stress (5)
- Famine/starvation (6)
- Poverty (7)
- Other (8)

End of Block: Trauma stressors

Start of Block: PTSD Symptoms

Q73
The following questions ask about different problems that people sometimes have. For each

one we would like to know how much you have experienced each one IN THE LAST MONTH, including today.

Q74 Repeated, disturbing memories, thoughts, or images of a stressful experience from the past?

- Not at all (1)
- A little bit (2)
- Moderately (3)
- Quite a bit (4)
- Extremely (5)

Q75 Feeling very upset when something reminded you of a stressful experience from the past?

- Not at all (1)
- A little bit (2)
- Moderately (3)
- Quite a bit (4)
- Extremely (5)

Q76
Avoid activities or situations because they remind you of a stressful experience from the past?

- Not at all (1)
- A little bit (2)
- Moderately (3)
- Quite a bit (4)
- Extremely (5)

Q77
Feeling distant or cut off from other people?

- Not at all (1)
- A little bit (2)
- Moderately (3)
- Quite a bit (4)
- Extremely (5)

Q78
Feeling irritable or having angry outbursts?

○ Not at all (1)

○ A little bit (2)

○ Moderately (3)

○ Quite a bit (4)

○ Extremely (5)

Q79
Having difficulty concentrating?

○ Not at all (1)

○ A little bit (2)

○ Moderately (3)

○ Quite a bit (4)

○ Extremely (5)

End of Block: PTSD Symptoms

Start of Block: Experiences of Family Violence

Q80 Now we would like to ask a few more questions about the types of violence that some people may experience at home. These questions will ask about three different types of violence: Physical, Emotional and Sexual.

Physical violence is when someone hits, slaps, kicks, punches or does anything else to hurt you

physically.

Emotional violence is when someone says or does something to humiliate you in front of others, threatens to harm you, or insults you and makes you feel bad about yourself.

Sexual violence is when someone forces you in any way to have sexual intercourse or perform any other sexual acts.

As always, your answers to these questions will be kept secret and will not be shared with anyone else.

X→

Q81 Have you ever been subject to violence (Physical, Emotional and Sexual) at home?

- ◯ Yes (1)
- ◯ No (2)

Skip To: Q84 If Have you ever been subject to violence (Physical, Emotional and Sexual) at home? = No

---

Display This Question:

If Have you ever been subject to violence (Physical, Emotional and Sexual) at home? = Yes

X→

Q82 Did this happen to you as an adult and/or as a child?

- ◯ Child (before age 18) (1)
- ◯ Adult (after age 18) (2)
- ◯ Both (before and after age 18) (3)

Display This Question:

If Have you ever been subject to violence (Physical, Emotional and Sexual) at home? = Yes

Q83 **What type of violence?**
(Select all that apply)

- ▢ Physical (hitting, slapping, kicking, or anything else to hurt you physically) (1)
- ▢ Emotional (humiliation, threats of harm, insults) (2)
- ▢ Sexual (forcing in any way to have sexual intercourse or perform any other sexual acts) (3)
- ▢ Other (4)

Q84 Have you ever subjected one of your family members to violence (Physical, Emotional, or Sexual) at home?

- ◯ Yes (1)
- ◯ No (2)

Skip To: End of Block If Have you ever subjected one of your family members to violence (Physical, Emotional, or Sexual) a... = No

---

Display This Question:

If Have you ever subjected one of your family members to violence (Physical, Emotional, or Sexual) a... = Yes

Q85 **What type of violence?**
(Select all that apply)

- ☐ Physical (hitting, slapping, kicking, or anything else to cause physical hurt) (1)
- ☐ Emotional (humiliation, threats of harm, insults) (2)
- ☐ Sexual (forcing in any way to have sexual intercourse or perform any other sexual acts) (3)
- ☐ Other (4)

End of Block: Experiences of Family Violence

Start of Block: Resilience factors

Q86 We know some of those were difficult questions to answer. Thank you for doing so, your answers are really important. The following questions will ask about your outlook on life, your social support networks, and your economic situation.

Q87
How often do you have someone to confide in or talk to about yourself or your problems?

- ○ Never (1)
- ○ Rarely (2)
- ○ Some of the time (3)
- ○ Most of the time (4)
- ○ All of the time (5)
- ○ Do not know (88)

Q88 **When I feel lonely, abandoned, sad, deeply upset, feel down, angry, frustrated for different reasons I seek support from:**
(Select all that apply)

- ☐ Family members or elders (1)
- ☐ Village elders (2)
- ☐ Peers with similar experience (3)
- ☐ Religious places/leaders (4)
- ☐ Professionals (Doctors, Counsellors, Lawyers Social Workers) (5)
- ☐ Other (6)
- ☐ I do not have any place to go (7)
- ☐ I do not seek support at all (8)
- ☐ Do not know (88)

Q89 How do you feel about your family's current financial situation?

- Very good (1)
- Good (2)
- A little hard (3)
- Very hard (4)
- Do not know (88)

Q90 How economically independent are you?

- Very Much (1)
- Somewhat (2)
- Not at all (3)
- Do not know (88)

Q91 How is your economic situation compared with most of your neighbors'?

- Much better (1)
- Somewhat better (2)
- Similar (3)
- Somewhat worse (4)
- Much worse (5)
- Do not know (88)

End of Block: Resilience factors

Start of Block: Peacebuilding attitudes

Q92 You are progressing very well in the survey, thank you. The next questions will ask about your attitudes towards different issues related to peace.

Q93 A truth-telling process aims to uncover the facts and context of what happened during past conflict or violence. In Nepal, three official mechanisms have been set in place (the Truth and Reconciliation Committee (TRC), the Commission of Investigation on Enforced Disappeared Person (CIEDP) , and the National Human Rights Commission). [EF1]

Thinking about these mechanisms, indicate how much you agree or disagree with the following statements.

Q94
It is better not to open up old wounds by talking about what happened in the past.

- Strongly agree (1)
- Somewhat agree (2)
- Somewhat disagree (3)
- Strongly disagree (4)
- Do not know (88)

Q95 It is important to know what happened during the conflict and collect testimonies.

- Strongly agree (1)
- Somewhat agree (2)
- Somewhat disagree (3)
- Strongly disagree (4)
- Do not know (88)

Q96 Sharing the truth about conflict experiences would help to build more positive relationships between members from *different* communities.

- Strongly agree (1)
- Somewhat agree (2)
- Somewhat disagree (3)
- Strongly disagree (4)
- Do not know (88)

Q97 Sharing the truth about conflict experiences would help to build more positive relationships between members *within my own* community.

- Strongly agree (1)
- Somewhat agree (2)
- Somewhat disagree (3)
- Strongly disagree (4)
- Do not know (88)

Q98 The next question will ask about your own experience with different commissions and mechanisms that have happened in Nepal, either in the past or that are now ongoing. Please answer from your own personal experience.

Q99 **Have you given testimony to any of the following commissions or mechanisms? (Select all that apply)**

- ☐ Truth and Reconciliation Commission (TRC) (1)
- ☐ Commission of Investigation on Enforced Disappeared Person (CIEDP) (2)
- ☐ Human Rights Commission (3)
- ☐ Office of the United Nations High Commissioner for Human Rights (OHCHR) (4)
- ☐ Conflict victims committees (5)
- ☐ Human Rights NGOs (6)
- ☐ Community-based indigenous organizations (7)
- ☐ Local Peace Committee (LPC) (8)
- ☐ Other (9)
- ☐ None of the above (10)

Skip To: Q101 If Have you given testimony to any of the following commissions or mechanisms? (Select all that apply) = None of the above

Q100 How satisfied are you with your experience of participating in this/these institution(s)?

- Extremely satisfied (1)
- Satisfied (2)
- Dissatisfied (3)
- Extremely dissatisfied (4)
- Mixed experiences (5)
- Do not know (88)

Q101 The next questions will ask your opinion on some common statements about yourself and society. For each statement, indicate how much you agree or disagree:

Q102 If I think I am right about something, I don't waste much time listening to other people's arguments.

- Strongly agree (1)
- Somewhat agree (2)
- Somewhat disagree (3)
- Strongly disagree (4)
- Do not know (88)

Q103 When I criticize someone, I think about how I would feel if I were in his/her place.

- Strongly agree (1)
- Somewhat agree (2)
- Somewhat disagree (3)
- Strongly disagree (4)
- Do not know (88)

Q104
Next, we will ask some questions about trust.

Generally speaking, would you say that most people can be trusted or that you need to be very careful in dealing with people?

- Most people can be trusted (1)
- Need to be very careful (2)
- Do not know (88)

Q105 Do you think one should always be on guard towards other people, even if they are neighbours or friends?6

- Yes (1)
- No (2)
- Do not know (88)

Q106 Do you think most people would try to take advantage of you if they got the chance, or would they try to be fair?

- Most people would try to take advantage of you (1)
- Most people would try to be fair (2)
- Do not know (88)

Q107 We will ask a few more questions regarding trust. Remember that everything you share here will only be used for research purposes and will be kept strictly secret.

Q108 I'd like to ask you how much you trust people from various groups. Could you tell me for each whether you trust people from...

	Very much (1)	A little (2)	Not at all (3)	Do not know (88)
Your family (Q84_1)	○	○	○	○
People of the same gender as yourself (Q84_2)	○	○	○	○
People of a different gender than yourself (Q84_3)	○	○	○	○
People in your neighborhood (Q84_4)	○	○	○	○
People of another ethnicity or caste (Q84_5)	○	○	○	○
People from other areas of Nepal (Q84_6)	○	○	○	○
Maoist members/leaders (Q84_7)	○	○	○	○
Members/leaders of other political parties (Q84_8)	○	○	○	○
Army/police force (Q84_9)	○	○	○	○
People from another country (Q84_10)	○	○	○	○

Q109 To what extent do you feel threatened when you are around people from the following groups?

	Very much (1)	A little (2)	Not at all (3)	Do not know (88)
Your family (Q85_1)	○	○	○	○
People of the same gender as yourself (Q85_2)	○	○	○	○
People of a different gender than yourself (Q85_3)	○	○	○	○
People in your neighborhood (Q85_4)	○	○	○	○
People of another ethnicity or caste (Q85_5)	○	○	○	○
People from other areas of Nepal (Q85_6)	○	○	○	○
Maoist members/leaders (Q85_7)	○	○	○	○
Members/leaders of other political parties (Q85_8)	○	○	○	○
Army/police force (Q85_9)	○	○	○	○
People from another country (Q85_10)	○	○	○	○

Q110 How comfortable would you feel in the following situations in the presence of a former member of the Maoist army?

	Completely comfortable (1)	Somewhat Comfortable (2)	Somewhat Uncomfortable (3)	Completely Uncomfortable (4)
Working with them (Q86_1)	◯	◯	◯	◯
Living in the same village/ community (Q86_2)	◯	◯	◯	◯
Living as close neighbors (Q86_3)	◯	◯	◯	◯
Living as a family member (Q86_4)	◯	◯	◯	◯
Marrying a family member (Q86_5)	◯	◯	◯	◯

Q111 Would you describe yourself as being a member of a group that is currently discriminated against or being treated unfairly in Nepal?

◯ Yes (1)

◯ No (2)

◯ Do not know (88)

Display This Question:

If Would you describe yourself as being a member of a group that is currently discriminated against... = Yes

Q112 **On what ground(s) is your group discriminated against? (Select all that apply)**

- ☐ Religion (1)
- ☐ Language (2)
- ☐ Ethnicity or caste (3)
- ☐ Regional basis (4)
- ☐ Economic background or class (5)
- ☐ Age (6)
- ☐ Gender (7)
- ☐ Sexual orientation (8)
- ☐ Disability (9)
- ☐ Other (10)
- ☐ Do not know (88)

Q113 Now we will turn to some other questions. To end the armed conflict in Nepal there were lengthy negotiations between the parties to the conflict before a peace agreement was signed in 2006. We would like to ask some questions about what you think about the peace agreement.

Q114 Have you heard about the Comprehensive Peace Accord 2006–Bistrit Shanti Samjhauta?

◯ Yes (1)

◯ No (2)

Display This Question:

If Have you heard about the Comprehensive Peace Accord 2006–Bistrit Shanti Samjhauta? = Yes

Q115 Please indicate how much you agree or disagree with the following statement: The peace agreement was necessary to end the armed conflict.

◯ Completely agree (1)

◯ Somewhat agree (2)

◯ Somewhat disagree (3)

◯ Completely disagree (4)

◯ Do not know (88)

Display This Question:

If Have you heard about the Comprehensive Peace Accord 2006–Bistrit Shanti Samjhauta? = Yes

Q116 Please indicate how much you agree or disagree with the following statement: The peace agreement reflects the will of the Nepali people.

- ◯ Completely agree (1)
- ◯ Somewhat agree (2)
- ◯ Somewhat disagree (3)
- ◯ Completely disagree (4)
- ◯ Do not know (88)

Display This Question:

If Have you heard about the Comprehensive Peace Accord 2006–Bistrit Shanti Samjhauta? = Yes

Q117 Please indicate how much you agree or disagree with the following statement: The peace agreement has not been implemented properly.

- ◯ Completely agree (1)
- ◯ Somewhat agree (2)
- ◯ Somewhat disagree (3)
- ◯ Completely disagree (4)
- ◯ Do not know (88)

Q118
We would now like to ask about your views on responsibility. Please indicate how much you agree or disagree with the following statements.

All those who perpetrated violence in the 1996-2006 armed conflict, regardless of side, should be held responsible.

- Completely agree (1)
- Somewhat agree (2)
- Somewhat disagree (3)
- Completely disagree (4)
- Do not know (88)

Q119 **Should any of the following things happen to those who perpetrated violence in the 1996-2006 conflict?**

(Select all that apply)

- ▢ Nothing should happen to them (1)
- ▢ Punish them (2)
- ▢ Put them in jail (3)
- ▢ See them in trials / court (4)
- ▢ Have them compensate victims (5)
- ▢ They should ask for forgiveness (6)
- ▢ They should confess their crimes (7)
- ▢ They should be forgiven (8)
- ▢ Give them amnesty (9)
- ▢ Reintegrate them in the community, if this has not happened in the past (10)
- ▢ Other (11)

Q120 Should any of the following things happen to civilians who provided food, shelter or information to the Maoist army or government security forces during the armed conflict 1996-2006?

(Select all that apply)

- ▢ Nothing should happen to them (1)
- ▢ Punish them (2)
- ▢ Put them in jail (3)
- ▢ See them in trials / court (4)
- ▢ Have them compensate victims (5)
- ▢ They should ask for forgiveness (6)
- ▢ They should confess their crimes (7)
- ▢ They should be forgiven (8)
- ▢ Reward/honor them (9)
- ▢ Reintegrate them in the community (10)
- ▢ Other (11)

Q121 We will now ask a few questions about reconciliation programs.

First, are you aware of any reconciliation programs or programs bringing people from the different warring sides together in Nepal?

- Yes (1)
- No (2)

Display This Question:

If We will now ask a few questions about reconciliation programs. First, are you aware of any recon... = Yes

X→

Q122 In general, how important would you say the programs were for reconciliation in Nepal? Would you say they were:

- Very important (1)
- Moderately important (2)
- Of little importance (3)
- Not at all important (4)
- Do not know (88)

Display This Question:

If We will now ask a few questions about reconciliation programs. First, are you aware of any recon... = Yes

X→

Q123 Have you participated in any such reconciliation programs?

- Yes (1)
- No (2)

Display This Question:

If Have you participated in any such reconciliation programs? = Yes

Q124 **What kind of program did you participate in? (Select all that apply)**

- ☐ Skill/vocational development training (1)
- ☐ Community-based interaction programs (2)
- ☐ Sports (3)
- ☐ Cultural programs (e.g. folk song competition) (4)
- ☐ Picnic (5)
- ☐ Repatriation program for conflict victims (6)
- ☐ Participation in the joint conflict victims' platforms (7)
- ☐ Other (8)

Display This Question:

If Have you participated in any such reconciliation programs? = Yes

Q125 In the programs that you participated in, how important would you say that the programs were for reconciliation in Nepal?

- ○ Very important (1)
- ○ Moderately important (2)
- ○ Of little importance (3)
- ○ Not at all important (4)
- ○ Do not know (88)

End of Block: Peacebuilding attitudes

Start of Block: Gender Equality Attitudes

Q126 You have now reached the final section of the survey, with a few questions about the roles of men and women. This is a different topic than our previous questions, but this topic is also important to our study.

Please tell us how much you agree with the following statements:

X→

Q127 A man shouldn’t show emotions and weakness.

- ○ Completely agree (1)
- ○ Somewhat agree (2)
- ○ Somewhat disagree (3)
- ○ Completely disagree (4)
- ○ Do not know (88)

Q128 It is manly to defend the honor of your family even by violent means.

- Completely agree (1)
- Somewhat agree (2)
- Somewhat disagree (3)
- Completely disagree (4)
- Do not know (88)

Q129 The male family members should make the final decision on all matters.

- Completely agree (1)
- Somewhat agree (2)
- Somewhat disagree (3)
- Completely disagree (4)
- Do not know (88)

Q130 There are times when a woman deserves to be beaten.

- Completely agree (1)
- Somewhat agree (2)
- Somewhat disagree (3)
- Completely disagree (4)
- Do not know (88)

Q131 Men are better suited to own land than women are.

- Completely agree (1)
- Somewhat agree (2)
- Somewhat disagree (3)
- Completely disagree (4)
- Do not know (88)

Q132 When jobs are scarce, men should have more right to a job than women.

- ○ Completely agree (1)
- ○ Somewhat agree (2)
- ○ Somewhat disagree (3)
- ○ Completely disagree (4)
- ○ Do not know (88)

X→

Q133 If a woman earns more money than her husband, it's almost certain to cause problems.

- ○ Completely agree (1)
- ○ Somewhat agree (2)
- ○ Somewhat disagree (3)
- ○ Completely disagree (4)
- ○ Do not know (88)

End of Block: Gender Equality Attitudes

Start of Block: End Survey

Q134
Thank you, the survey is now finished! We greatly appreciate your participation. Please hand the device over to the interviewer.

End of Block: End Survey

Start of Block: Validation

Q135 Enter password

____

Display This Question:

If If Enter password Text Response Is Not Equal to 1234

Q136 I'm sorry, that ID number was incorrect. Please use the back button to return to the last question and re-enter the correct ID number.

End of Block: Validation

Start of Block: Post-Survey Observation

Q137 Interview privacy; the respondent was left in privacy with no interruptions while tapping in answers.

○ Yes (1)

○ No (2)

Q138 PDA Use

○ Self-administered (1)

○ Partially self-administered, some assistance (2)

○ Enumerator assisted respondent throughout the survey (3)

Q139 Did the respondent ask for assistance mid-interview?

- Yes (1)
- No (2)

Skip To: Q141 If Did the respondent ask for assistance mid-interview? = No

Display This Question:

If Did the respondent ask for assistance mid-interview? = Yes

Q140 What type of assistance? (Select all that apply)

- Technical assistance (related to the tablet) (1)
- Substantial understanding of the survey questions (2)
- Financial assistance (3)
- Other material assistance (4)
- Other (5)

Q141 As far as you can tell, did the respondent have an emotional reaction to the questions?

- Yes (1)
- No (2)

Skip To: Q143 If As far as you can tell, did the respondent have an emotional reaction to the questions? = No

Display This Question:

If As far as you can tell, did the respondent have an emotional reaction to the questions? = Yes

Q142 Please specify

____

Q143 Is there anything else to note?

____

End of Block: Post-Survey Observation
