# Supplementary material for: Women, peace and insecurity: The risks of peacebuilding in everyday life for women in Sri Lanka and Nepal
Source: PLoS One. 2024 May 29;19(5):e0303023. doi: 10.1371/journal.pone.0303023 (PMC11135728; doi:10.1371/journal.pone.0303023)
Supplement: S1 Appendix — (PDF) [file pone.0303023.s004.pdf]

Women, Peace and Insecurity: The risks of peacebuilding in everyday life for women in Sri Lanka and Nepal

Appendix

Survey information 2

Sri Lanka 3

Nepal 4

Measurements of variables used in article 6

Coexistence and trust 6

Sri Lanka 6

Nepal 7

Truthtelling 8

Sri Lanka 8

Nepal 9

Accountability 10

Sri Lanka 10

Nepal 10

Peace accord (Nepal only) 10

Control variables 11

Descriptive data (all estimations in attached do-files) 14

Coexistence and trust (by gender) 14

Truthtelling (by gender) 16

Accountability (by gender) 16

Peace accord, by gender (Nepal only) 17

Control variables 18

Sri Lanka 18

Nepal 18

Robustness checks (all estimations in attached do-files) 19

Alternative operationalizations of main dependent variables 19
Coexistence and trust – Sri Lanka and Nepal 19
Truthtelling – Sri Lanka and Nepal 21
Accountability – Sri Lanka and Nepal 21
Peace accord views (Nepal) 23
Additional control variables 23
Trust and coexistence – Sri Lanka 24
Trust and coexistence – Nepal 25
Truthtelling – Sri Lanka and Nepal 26
Accountability – Sri Lanka and Nepal 27
Peace accord views (Nepal) 28
Trimmed models 28
Trust and coexistence – Sri Lanka 29
Trust and coexistence – Nepal 30
Truthtelling – Sri Lanka and Nepal 30
Accountability – Sri Lanka and Nepal 31
Peace accord views (Nepal) 32

Survey information

The survey was developed as part of the *Gender, War trauma and Peacebuilding project*, funded by the Swedish Research Council (grant number 2014-03780). Both the survey and the focus group interviews that were conducted in parallel were approved by the Swedish Ethical Review Authority (Dnr 2016/342 and Dnr 2016/552), and by authorities in Sri Lanka and Nepal at the national, regional, and local level, in accordance with local procedure.

The survey questionnaire was developed through an iterative process, where the draft was translated, back-translated, piloted, re-phrased and finalized, in continuous dialogue between the research partners in Uppsala (Department of Peace and Conflict Research, Uppsala University), Kathmandu (Center for Social Change) and Colombo (University of Colombo). The final questionnaire had seven sections, asking questions on: demographics, trauma

stressors, PTSD symptoms, experiences of family violence, resilience factors, peacebuilding attitudes, and gender equality attitudes. While we aimed to streamline the questions across the two countries, they were also designed and adapted to fit each context (Sri Lanka and Nepal); hence, the inclusion and wording of some questions differ across each case. In both countries, the interviewer filled in a few pre-survey observations on location (in Sri Lanka: district, DS number, and GN number; in Nepal: district, municipality/VDC, and ward), household number and the respondent's gender before the actual survey began, and asked the interviewee for their consent to participate, as well as informed them that they could choose to decline or discontinue the interview at any time. The survey data was collected using Personal Digital Assistants (PDAs, i.e. tablets) to maximize anonymity and minimize known interviewer-effects such as social desirability bias and acquiescence. However, many respondents opted to have the enumerators assist them in typing in their responses.[1] Post-survey, the enumerator filled out information about whether the respondent was left in privacy while tapping in questions, if they requested assistance with the PDA or other types of assistance, and whether the respondent had an emotional reaction to the questions.

As will be described in more detail below, the sampling procedures differed in the two countries, to tailor to the specific circumstances of each country's conflict background, and to ensure our study would be meaningful to each context. It should be noted that neither sample aspires to be nationally representative; hence, the results cannot be generalized to the whole population and the results for the two countries are reported separately rather than pooling the data. However, findings of this study are relevant at least in areas similar to the study districts, and can open to interesting questions for each country as a whole.

Sri Lanka

In Sri Lanka, the survey study was carried out between October 27 to November 15, 2017. In total, 1,028 respondents (50% women, 50% men) were interviewed for the survey. The survey was carried out in three districts to reach populations affected by the Sri Lankan war, while ensuring equal distribution between the three ethnic communities: Anuradhapura (Sinhalese respondents, N: 341), Vavuniya (Tamil respondents, N: 346) and Mannar (Muslim respondents, N: 341). In each of the three districts, four to five DS divisions were sampled.

[1] In Nepal, the majority (61%) chose to be assisted by enumerators through the whole questionnaire, another 10% was partially assisted, while 29% fully self-administered. In Sri Lanka, the corresponding numbers were 48% (assisted fully), 24% (assisted partially) and 28% (self-administered).

The study spanned the entire districts of Mannar and Vavuniya, while in the Anuradhapura district, questionnaires were administered only among the Divisional Secretariat Divisions (DSDs) which were border villages. This ensured that the survey was largely carried out among groups of persons who had experiences relevant to the focus of the study.

Villages in each district were chosen according to probability proportionate to size (PPS) sampling. Within each district, 17 starting points (for this study: a small grocery shop or school) were distributed according to their population proportion. Within each starting point, 20 interviews were conducted. From the starting point, households were chosen using the right-hand rule and a two-household skip pattern. Respondents within a household were chosen through the 'last birthday' method. The sample size per district, by gender, was as follows:

**District**	**Sample**	**Women**	**Men**
Vavuniya	346	173	173
Anuradhapura	341	173	168
Mannar	341	173	168
Total	*1028*	*519*	*509*

The enumerators – three teams of ten enumerators, with two supervisors leading each team – were recruited and instructed by Dr Pradeep Peiris and his group at the Social Scientists' Association (SSA), Colombo. A three-day training of the enumerators was held at the University of Colombo on October 22-24, 2017, on, for example, practicing to use PDAs for data collection, understanding the survey questionnaire itself, discussing difficulties in field work and field work ethics, and the sampling technique of this study. On average, each survey took one hour to complete in the field.

Nepal

In Nepal, the survey data was collected 3 February – 3 March, 2018. It included 1,013 respondents (50% women, 50% men) from six districts throughout the country. The six districts were selected and categorized based on conflict intensity, with three districts from high conflict areas (HCA) and three districts from low conflict areas (LCA). Conflict intensity was defined on the basis of district specific killings, disappearances, and displacement cases recorded through Informal Sector Service Centre (INSEC), a Kathmandu-based human rights

organization who regularly records human rights violation cases across the country. This selection was also informed by the Government of Nepal's categorization of districts based on the intensity and impact of the armed conflict. The selected LCA districts were Sunsari, Morang, and Jhapa in the Eastern part of Nepal and the HCA districts were Bardiya, Surkhet, and Dang in the Mid-Western part of Nepal. In each district, municipalities/VDCs, wards, and small settlements within each ward were selected randomly, proportional to population size and gender balance.

Respondents from HCA districts were slightly oversampled and constitute around 60% of respondents in the Nepal study; the remaining 40% were from LCA districts. The sample size per district, by gender, was as follows:

**District**	**Sample**	**Women**	**Men**
Sunsari	102	51	51
Morang	150	76	74
Jhapa	150	75	75
Bardiya	201	100	101
Surkhet	203	102	101
Dang	207	102	105
Total	*1013*	*506*	*507*

The enumerators were recruited in the six sampled districts and participated in a three-day training on research and ethics conducted by the joint research team (Uppsala, Kathmandu and Colombo) 5-7 September 2017, prior to the implementation of the survey. The training e.g. included practicing to use PDAs, discussing the survey questions, the sampling technique as well as crucial questions related to safety, ethics, and best practice during fieldwork. The fieldwork was coordinated by Dr Prakash Bhattarai at CSC, Kathmandu. On average, each survey took one hour to complete.

Measurements of variables used in article

All variables used in this article is also described in the questionnaires, which are provided in full as part of the supplementary files. To facilitate the analysis and readability in this articles, some scales have been reversed to make them more intuitive in the article manuscript (i.e. more of something – e.g more perceived trust, or how much one agrees with specific statements – has a higher value). The scales reported below are those which are used in the statistical analysis performed in this article.

The below figure summarizes the dependent variables, in groups following the theoretical expectations:

		**Sri Lanka**	**Nepal**
Affecting daily lives:<br>**Gender difference expected**	Coexistence and trust	• Trust in Sinhala<br>• Trust in Tamil<br>• Trust in Muslim<br>• Feel threatened around Sinhala<br>• Feel threatened around Tamil<br>• Feel threatened around Muslim<br>• Feel comfortable around Army<br>• Feel comfortable around ex-LTTE	• Trust in Army<br>• Trust in Maoists<br>• Feel threatened around Army<br>• Feel threatened around Maoists<br>• Feel comfortable around Maoists
	Truthtelling	• Important to collect testimonies<br>• Sharing truth build positive relationships	• Important to collect testimonies<br>• Sharing truth build positive relationships
Elite initiatives:<br>**No gender difference expected**	Accountability	• Perpetrators should be held responsible	• Perpetrators should be held responsible
	Peace accord	N/A	• The CPA was necessary to end conflict<br>• The CPA reflects the will of the Nepali people

Coexistence and trust

Sri Lanka

For the variables *Trust in Sinhala*, *Trust in Tamil*, and *Trust in Muslim* the following question is used:

"I'd like to ask you how much you trust people from various groups. Could you tell me for each whether you trust people from…[*Sinhala people*]; [*Tamil people*]; [*Muslim people*]

(0) Not at all
(1) A little
(2) Very much

Estimated using ordinal logistic regression.

For the variables *Threatened around Sinhala*, *Threatened around Tamil*, and *Threatened around Muslim* the following question is used:

"To what extent do you feel threatened when you are around people from the following groups? [*Sinhala people*]; [*Tamil people*]; [*Muslim people*]

(0) Not at all
(1) A little
(2) Very much

Estimated using ordinal logistic regression.

For the variables *Feel comfortable around Army* and *Feel comfortable around ex-LTTE* the following question is used:

"How comfortable would you feel in the following situations in the presence of current Sri Lanka Army soldiers/a former member of the LTTE" For each of the following items: "Working with them", "Living in the same village/community", "Living as close neighbors", and "Marrying a family member", the respondents answer on the following scale:

(0) Completely uncomfortable
(1) Somewhat uncomfortable
(2) Somewhat comfortable
(3) Completely comfortable

In the reported analysis, a composite measure combining the responses on all items (the different hypothetical situations described above) is used, ranging from 0 to 12, and estimated using OLS regression. In alternative tests, each item was also tested separately (see below).

Nepal

For the variables *Trust in Army* and *Trust in Maoists*, the following questions are used:

"I'd like to ask you how much you trust people from various groups. Could you tell me for each whether you trust people from…[*The army*]; [*Maoists*]

(0) Not at all
(1) A little
(2) Very much

Estimated using ordinal logistic regression.

For the variables *Feel threatened around Army* and *Feel threatened around Maoists* the following question is used:

"To what extent do you feel threatened when you are around people from the following groups?" [*Army*]; [*Maoist members/leaders*]

(0) Not at all
(1) A little
(2) Very much

Estimated using ordinal logistic regression.

For the variable *Feel comfortable around Maoists* the following question is used:

"How comfortable would you feel in the following situations in the presence of a former member of the Maoist army" For each of the following items: "Working with them", "Living in the same village/community", "Living as close neighbors", "Living as a family member" and "Marrying a family member", the respondents answer on the following scale:

(0) Completely uncomfortable
(1) Somewhat uncomfortable
(2) Somewhat comfortable
(3) Completely comfortable

In the reported analysis, a composite measure combining the responses on all items is used, ranging from 0 to 15, and estimated using OLS regression. In alternative tests, each item was also tested separately (see below).

Truthtelling

Sri Lanka

For the variable *Important to collect testimonies*, the following question is used:

"It is important to know what happened during the conflict and collect testimonies"

(0) Strongly disagree

(1) Somewhat disagree
(2) Somewhat agree
(3) Strongly agree

Estimated using ordinal logistic regression.

For the variable *Sharing truth build positive relationships*, the following question is used:

"Sharing the truth about conflict experiences would help build more positive relationships between my ethnic group and different ethnic groups"

(0) Strongly disagree
(1) Somewhat disagree
(2) Somewhat agree
(3) Strongly agree

Estimated using ordinal logistic regression.

Nepal

For the variable *Important to collect testimonies*, the following question is used:

"It is important to know what happened during the conflict and collect testimonies"

(0) Strongly disagree
(1) Somewhat disagree
(2) Somewhat agree
(3) Strongly agree

Estimated using ordinal logistic regression.

For the variable *Sharing truth build positive relationships*, the following question is used:

"Sharing the truth about conflict experiences would help build more positive relationships between members from different communities"

(0) Strongly disagree
(1) Somewhat disagree
(2) Somewhat agree
(3) Strongly agree

Estimated using ordinal logistic regression.

Accountability

Sri Lanka

For the variable *Perpetrators should be held responsible*, the following question is used:

"Please indicate how much you agree or disagree with the following statements: All those that perpetrated violence in the 1983-2009 conflict, regardless of side, should be held responsible"

(0) Strongly disagree
(1) Somewhat disagree
(2) Somewhat agree
(3) Strongly agree

Estimated using ordinal logistic regression.

Nepal

For the variable *Perpetrators should be held responsible*, the following question is used:

"Please indicate how much you agree or disagree with the following statements: All those that perpetrated violence in the 1996-2006 armed conflict, regardless of side, should be held responsible"

(0) Strongly disagree
(1) Somewhat disagree
(2) Somewhat agree
(3) Strongly agree

Estimated using ordinal logistic regression.

Peace accord (Nepal only)

For the variable *The CPA was necessary to end conflict*, the following question is used:

"Please indicate how much you agree or disagree with the following statements: The peace agreement was necessary to end the armed conflict"

(0) Strongly disagree
(1) Somewhat disagree
(2) Somewhat agree

(3) Strongly agree

Estimated using ordinal logistic regression.

For the variable *The CPA reflects the will of the Nepali people*, the following question is used:

"Please indicate how much you agree or disagree with the following statements: The peace agreement reflects the will of the Nepali people"

(0) Strongly disagree
(1) Somewhat disagree
(2) Somewhat agree
(3) Strongly agree

Estimated using ordinal logistic regression.

Control variables

Affected by conflict, counts the number of types of conflict exposure the respondent reported being exposed to among the following:

- ☐ Displacement
- ☐ Belongings stolen
- ☐ Belongings destroyed
- ☐ House destroyed
- ☐ House/land seized
- ☐ Forced donation (e.g. monthly levy)
- ☐ Threatened with violence
- ☐ Threatened with death
- ☐ Witnessed violence
- ☐ Beaten violently or tortured
- ☐ Forced to commit violence
- ☐ Experienced sexual violence or sexual abuse
- ☐ Witnessed sexual violence or sexual abuse
- ☐ Forced to commit sexual violence or sexual abuse
- ☐ Family member (Spouse/Child/Parent) injured
- ☐ Family member (Spouse/Child/Parent) killed
- ☐ Family member (Spouse/Child/Parent) abducted

- ▢ Family member (Spouse/Child/Parent) disabled
- ▢ Saw your loved ones dying in front of you
- ▢ Imprisoned
- ▢ Injured by land mines
- ▢ Wounded due to shell attacks/ bomb blasts/ unexpected attacks

The variable hence theoretically spans from 0 to 22, but the highest number reported in the data is 17 for Nepal and 20 for Sri Lanka.

To control for the respondent's mental health, we include a binary measure for symptoms of *Posttraumatic Stress Disorder* (*PTSD*). While assessing PTSD across cultures is tricky it can give an indication of the magnitude of war-related trauma in a population, and its accompanying difficulties after war. To minimize exposure to difficult memories, we asked the short 6-question measure of PTSD previously used in war-affected settings, using the standard cut-off rates. Each question ranges from 1 (not at all) to 5 (extremely) and those that sums to 14 or above are considered reaching the threshold (coded 1; otherwise 0).

(0) no PTSD
(1) PTSD)

We further control for the respondent's *Physical health* using this question: "Thinking about your general physical health [things like: sickness, illness, injury, disease etc.] – on a scale from 1, very poor, to 4, very good, how would you describe your overall physical health today?"

(1) Very Poor
(2) Somewhat Poor
(3) Somewhat Good
(4) Very Good

To control for *Education*, a dichotomous variable was created based on the responses to "What is the highest education level you have achieved?"

<u>Sri Lanka:</u>

- No formal school OR
- Between grades 1-5

coded (0) and

- Between grades 6-11,
- Up to Advanced level,
- Bachelor's Degree, OR
- Master's degree or professional degree

coded (1)

<u>Nepal:</u>

- No formal school OR
- Primary school

coded (0) and

- Secondary school,
- High School,
- Bachelor's degree,
- Master's degree or PhD, OR
- Professional degree

coded (1)

To control for *Family finance*, the following question is used: "How do you feel about your family's current financial situation?"

(0) Very hard
(1) A little hard
(2) Good
(3) Very good

In Nepal, we control for whether or not the respondent lives in a *High conflict area*, hence coded

(0) For respondents in Sunsari, Morang and Jhapa
(1) For respondents in Dang, Bardiya and Surkhet

For Sri Lanka ethnicity dummies are included ("Muslim" and "Tamil"; Sinhalese as reference category).

Descriptive data (all estimations in attached do-files)

Below, the frequency proportions of each of the variables presented in the previous section are outlined, per country and by gender.

Coexistence and trust (by gender)

First, we explore how respondents feel about coexisting and trust, starting with Sri Lanka. Here we include questions about trust and threat in relation to different ethnic groups, as well as how comfortable respondents feel around members of the army and former LTTE cadres. We start by reporting descriptive data by gender. Significant gender differences are marked with asterisk*

Sri Lanka

		**Not at all**	**A little**	**Very much**
Trust in Sinhala** (N: 554)	Women	80 (30%)	149 (55%)	41 (15%)
	Men	62 (22%)	186 (65%)	36 (13%)
	Total	142 (26%)	335 (60%)	77 (14%)
Trust in Tamil*** (N: 561)	Women	95 (34%)	148 (53%)	34 (12%)
	Men	55 (19%)	187 (66%)	42 (15%)
	Total	150 (27%)	335 (60%)	76 (14%)
Trust in Muslim** (N: 527)	Women	107 (42%)	132 (52%)	13 (5%)
	Men	85 (31%)	169 (61%)	21 (7%)
	Total	192 (36%)	301 (57%)	34 (6%)
Threat Sinhala (N: 595)	Women	107 (36%)	125 (42%)	67 (22%)
	Men	115 (39%)	116 (39%)	65 (22%)
	Total	222 (37%)	241 (41%)	132 (22%)
Threat Tamil (N: 577)	Women	139 (47%)	125 (42%)	33 (11%)
	Men	131 (47%)	122 (44%)	27 (10%)
	Total	270 (47%)	247 (43%)	60 (10%)
Threat Muslim (N: 537)	Women	116 (43%)	119 (44%)	37 (14%)
	Men	123 (46%)	104 (39%)	37 (14%)
	Total	239 (45%)	223 (42%)	75 (14%)
Comfortable army* (N: 997)	Women	Mean: 5.64 [5.31-5.96]		
	Men	Mean: 6.33 [6.01-6.65]		
Comfortable LTTE (N: 995)	Women	Mean: 3.48 [3.19-3.77]		
	Men	Mean: 4.04 [3.73-4.34]		

*gender difference is significant at 90% level; ** at 95% level; *** at 99% level.

As demonstrated above, women report significantly lower interethnic trust compared to men. There is no gender difference with regards to threat perceptions. In addition to trust and threat, the survey also asked how comfortable respondents would be around army soldiers and ex-LTTE cadres/members respectively, in a set of situations (for more information, see p 6 above). The composite measure varies from 0 (most uncomfortable) to 12 (most comfortable) and as can be seen in table above, women are significantly less comfortable than men around army soldiers (women's average is 5,6 and men's average is 6,3) while there is no significant gender difference regarding being around ex-LTTE (women's average 3,5, men's average 4,0).

Next, we look at frequency data related to measures of coexistence and trust in Nepal, with reference to conflict parties.

Nepal

		**Not at all**	**A little**	**Very much**
Trust in Maoists**	Women	205 (46%)	205 (46%)	38 (8%)
	Men	211 (46%)	187 (40%)	64 (14%)
	Total	416 (46%)	392 (43%)	102 (11%)
Trust in Army***	Women	121 (26%)	277 (60%)	63 (14%)
	Men	130 (28%)	214 (46%)	124 (27%)
	Total	251 (27%)	491 (53%)	187 (20%)
Feel threatened around Maoists***	Women	233 (50%)	160 (34%)	(73 (16%)
	Men	307 (67%)	115 (25%)	39 (8%)
	Total	540 (58%)	275 (30%)	112 (12%)
Feel threatened around Army***	Women	235 (49%)	181 (38%)	59 (12%)
	Men	307 (66%)	122 (26%)	38 (8%)
	Total	542 (58%)	303 (32%)	97 (10%)
Comfortable Maoists***	Women	Mean: 8.42 [8.07-8.76]		
	Men	Mean: 9.49 [9.16-9.81]		

*gender difference is significant at 90% level; ** at 95% level; *** at 99% level.

As indicated in these descriptive data, trust levels are generally quite low in Nepal. Additionally, women are significantly less trusting and feel more threatened by former conflict parties than men. A fifth measure for coexistence and trust is to what extent the respondents feel comfortable around Maoists in a set of circumstances. This variable ranges from 0 to 15 (more information how it was constructed is found above). Women are

significantly less comfortable around Maoists than men (women's average 8,4, men's average 9,5).

Truthtelling (by gender)

Below we report frequency data related to measures of truthtelling, i.e. views on the importance of collecting testimonies and whether respondents are positive to sharing the truth about the past conflict.

			**Completely disagree**	**Somewhat disagree**	**Somewhat agree**	**Completely agree**
Important to collect testimonies	Sri Lanka	Women	69 (14%)	34 (7%)	148 (31%)	230 (48%)
		Men	75 (16%)	37 (8%)	143 (30%)	221 (46%)
		Total	144 (15%)	71 (7%)	291 (30%)	451 (47%)
	Nepal**	Women	43 (10%)	23 (5%)	88 (20%)	288 (65%)
		Men	27 (6%)	14 (3%)	83 (18%)	343 (73%)
		Total	70 (8%)	37 (4%)	171 (19%)	631 (69%)
Positive to share truth	Sri Lanka**	Women	55 (11%)	39 (8%)	189 (39%)	201 (42%)
		Men	58 (12%)	23 (5%)	163 (34%)	237 (49%)
		Total	113 (12%)	62 (6%)	352 (36%)	438 (45%)
	Nepal	Women	29 (7%)	14 (3%)	125 (29%)	263 (61%)
		Men	20 (4%)	19 (4%)	123 (27%)	296 (65%)
		Total	49 (6%)	33 (4%)	248 (28%)	559 (63%)

*gender difference is significant at 90% level; ** at 95% level; *** at 99% level.

Starting with Sri Lanka, the table shows that both women and men find it important to collect testimonies, with no gender difference. Furthermore, the respondents are generally positive to sharing the truth – men significantly more so than women.

As for Nepal, the results are the other way around. Both women and men find it important to collect testimonies, but men significantly more so. Additionally, the respondents are positive to sharing the truth, with no significant gender difference.

Accountability (by gender)

Next, we report the frequency distributions regarding accountability based on the question whether perpetrators should be held responsible.

			**Completely disagree**	**Somewhat disagree**	**Somewhat agree**	**Completely agree**
Perpetrators should be held accountable	Nepal	Women	35 (8%)	22 (5%)	79 (19%)	283 (68%)
		Men	35 (8%)	20 (4%)	85 (19%)	309 (69%)
		Total	70 (8%)	42 (5%)	164 (19%)	592 (68%)
	Sri Lanka	Women	39 (8%)	24 (5%)	119 (25%)	299 (62%)
		Men	45 (10%)	28 (6%)	91 (19%)	306 (65%)
		Total	84 (9%)	51 (5%)	210 (22%)	605 (64%)

*gender difference is significant at 90% level; ** at 95% level; *** at 99% level.

The frequency distributions show support for holding perpetrators accountable in both countries, with no statistically significant gender difference.

Peace accord, by gender (Nepal only)

Last, we do not expect a gender difference in relation to how the peace agreement in Nepal is perceived. Cross-tabs of these views are reported here:

		**Completely disagree**	**Somewhat disagree**	**Somewhat agree**	**Completely agree**
CPA necessary	Women	0 (0%)	2 (1%)	15 (10%)	124 (88%)
	Men	7 (2%)	4 (1%)	22 (8%)	251 (88%)
	Total	7 (2%)	6 (1%)	37 (9%)	375 (88%)
CPA reflects will of Nepali people	Women	3 (2%)	3 (2%)	38 (28%)	90 (67%)
	Men	8 (3%)	9 (3%)	68 (24%)	198 (70%)
	Total	11 (3%)	12 (3%)	106 (25%)	288 (69%)

As can be seen in the table above, there is generally high approval of the CPA among the respondents: a very large majority agrees both that the agreement was necessary to end the conflict and that it reflects the will of the Nepali people. There is no gender difference, as expected from the hypothesis. Note that these questions were only asked to those respondents that answered affirmatively to the question whether they had heard of the CPA (43%, or 435 respondents).

Control variables

The tables below report summary statistics for control variables used in the analyses of the Sri Lanka and Nepal data.

Sri Lanka

Variable	N	Mean	Std.dev	Min	Max
affected	1021	4.364	3,429	0	20
ptsd14	1018	0.416	0.493	0	1
physhealth	1021	3.029	0.982	1	4
education	1021	0.792	0.406	0	1
famfinance	1018	2.194	0.804	1	4
muslim	1021	0.330	0.470	0	1
tamil	1021	0.337	0.473	0	1
sinhala	1021	0.333	0.472	0	1

Nepal

Variable	N	Mean	Std.dev	Min	Max
affected	1013	1.785	2.584	0	17
ptsd14	1010	0.324	0.468	0	1
physhealth	1011	3.043	0.976	1	4
education	1007	0.542	0.498	0	1
famfinance	1001	2.578	0.735	1	4
highconflict	1013	0.603	0.489	0	1

A few observations about these descriptives: the results indicate that the population in Sri Lanka was affected to a higher extent by the armed conflict and that more reach the clinical threshold for PTSD. More respondents have received basic education in Sri Lanka than in Nepal. The data from the two countries are similar in terms of the respondents' physical health and family income (Nepal slightly lower).

Robustness checks (all estimations in attached do-files)

Alternative operationalizations of main dependent variables

Coexistence and trust – Sri Lanka and Nepal

In the article, our main focus related to trust in Sri Lanka was trust in other ethnic groups and for Nepal trust in former conflict parties. Generally, women reported lower levels of trust in these groups. We also tested a measure for generalized trust, with the often-used question: "Generally speaking, would you say that most people can be trusted or that you need to be very careful in dealing with people". The pattern remains: women report significantly less trust than men, in both countries.

	(1)<br>Sri Lanka	(2)<br>Nepal
female	-0.486***	-0.623***
	(0.184)	(0.196)
affected	-0.058*	0.015
	(0.0322)	(0.039)
physhealth	0.196*	0.017
	(0.106)	(0.105)
ptsd14	-0.318	-0.036
	(0.203)	(0.217)
education	-0.342	-0.692***
	(0.233)	(0.194)
famfinance	-0.0411	0.102
	(0.120)	(0.137)
muslim	0.665**	
	(0.267)	
tamil	1.219***	
	(0.262)	
highconflict		-0.127
		(0.195)
constant	2.046***	-1.444***
	(0.457)	(0.471)
Observations	989	965

Standard errors in parentheses; *** $p<0.01$, ** $p<0.05$, * $p<0.1$

Examining the frequency distribution of this variable one can also conclude that trust levels are low in the examined in the population in both countries: in Sri Lanka, 88% of women and 81% of men answered No. In Nepal, 89% of women and 83% of men answered No.

Regarding coexistence in Sri Lanka, the main measures reported in the article - *Feel comfortable around ex-LTTE* and *Feel comfortable around army soldiers* – are composed by combining responses on how comfortable respondents are around ex-LTTE/army in four hypothetical scenarios (for more information, see discussion in the section "Measurement of

variables used in article"). When each component is tested separately, the results were the same (women significantly less comfortable) with regards to army soldiers, but less robustly so with regards to former LTTE members/cadres.

	Former LTTE				Army soldiers			
	(1) Work with	(2) Live same village	(3) Have as neighbor	(4) Marry	(5) Work with	(6) Live same village	(7) Have as neighbor	(8) Marry
female	-0.197	-0.203*	-0.201	-0.195	-0.507***	-0.298**	-0.335***	-0.330**
	(0.122)	(0.123)	(0.124)	(0.134)	(0.125)	(0.123)	(0.122)	(0.133)
affected	0.063***	0.045**	0.046**	0.035	-0.005	-0.049**	-0.034	-0.040*
	(0.021)	(0.021)	(0.022)	(0.023)	(0.021)	(0.021)	(0.021)	(0.024)
physhealth	0.017	0.007	0.017	-0.062	-0.080	-0.008	-0.047	-0.009
	(0.068)	(0.069)	(0.070)	(0.075)	(0.071)	(0.068)	(0.068)	(0.074)
ptsd14	-0.113	-0.088	-0.165	0.306**	0.114	0.042	0.032	0.279*
	(0.137)	(0.137)	(0.139)	(0.149)	(0.138)	(0.136)	(0.135)	(0.151)
education	0.339**	0.260	0.328**	0.361**	0.103	0.012	-0.025	-0.303*
	(0.163)	(0.162)	(0.164)	(0.181)	(0.164)	(0.161)	(0.159)	(0.173)
famfinance	0.059	0.040	0.095	0.121	-0.010	0.050	0.032	0.090
	(0.082)	(0.081)	(0.083)	(0.090)	(0.084)	(0.082)	(0.081)	(0.087)
muslim	-0.457***	-0.134	0.013	-0.304	-2.598***	-2.171***	-2.226***	-3.099***
	(0.173)	(0.171)	(0.174)	(0.195)	(0.185)	(0.178)	(0.176)	(0.200)
tamil	1.241***	1.712***	1.862***	1.046***	-3.071***	-2.559***	-2.455***	-3.509***
	(0.171)	(0.175)	(0.180)	(0.187)	(0.196)	(0.188)	(0.186)	(0.214)
/cut1	0.733**	0.688**	1.043***	1.424***	-3.530***	-3.203***	-3.172***	-2.813***
	(0.298)	(0.298)	(0.304)	(0.328)	(0.328)	(0.318)	(0.314)	(0.337)
/cut2	1.518***	1.499***	1.816***	2.402***	-2.733***	-2.382***	-2.283***	-1.999***
	(0.302)	(0.301)	(0.308)	(0.335)	(0.323)	(0.312)	(0.307)	(0.330)
/cut3	2.847***	2.948***	3.265***	3.505***	-1.339***	-0.864***	-0.911***	-0.502
	(0.314)	(0.315)	(0.323)	(0.350)	(0.312)	(0.303)	(0.299)	(0.319)
Observations	992	991	988	990	994	990	990	992

Standard errors in parentheses
*** p<0.01, ** p<0.05, * p<0.1

Regarding coexistence in Nepal, the main measure - *Feel comfortable around Maoists* - is a composite measure combining responses on how comfortable respondents are around a former member of the Maoist army in five hypothetical scenarios, ranging from 0 to 15. When each component is tested separately, the results were the same (women significantly less comfortable), as seen here:

	(1) Work with	(2) Live same village	(3) Have as neighbor	(4) Family member	(5) Marry
female	-0.144**	-0.291***	-0.270***	-0.120*	-0.185***
	(0.067)	(0.064)	(0.063)	(0.070)	(0.071)
affected	0.032**	0.025*	0.022*	0.041***	0.032**
	(0.014)	(0.013)	(0.013)	(0.014)	(0.014)
ptsd14	-0.110	-0.092	-0.003	-0.050	-0.141*
	(0.075)	(0.071)	(0.070)	(0.078)	(0.080)
physhealth	-0.018	0.007	0.007	-0.014	-0.025
	(0.037)	(0.035)	(0.035)	(0.039)	(0.039)
education	-0.217***	-0.201***	-0.190***	-0.104	-0.199***
	(0.068)	(0.064)	(0.063)	(0.070)	(0.072)
famfinance	-0.029	-0.088*	-0.043	0.002	-0.011
	(0.047)	(0.045)	(0.044)	(0.049)	(0.050)
highconflict	-0.092	-0.070	-0.069	-0.104	-0.125*
	(0.069)	(0.065)	(0.064)	(0.071)	(0.073)
Constant	2.159***	2.418***	2.264***	1.941***	1.904***
	(0.167)	(0.159)	(0.156)	(0.173)	(0.177)
Observations	983	981	980	978	975
R-squared	0.024	0.041	0.033	0.016	0.025

Standard errors in parentheses; *** p<0.01, ** p<0.05, * p<0.1

Truthtelling – Sri Lanka and Nepal

In Sri Lanka, we did not include any additional questions to capture attitudes related to truthtelling. However, we did ask whether respondents had given testimony in a range of commissions or mechanisms and those who answered affirmatively were asked a follow-up question about how satisfied they were with their experience of giving testimony. The results show that ca 12% of respondents (no gender difference) had given testimony. About half of them (52% of men; 41% of women, not a significant difference) reported to be satisfied or extremely satisfied with the experience; the remaining half were either dissatisfied, extremely dissatisfied or had mixed experiences.

Similarly, in Nepal, we also asked about personal experiences of given testimony in a commission or other type of mechanism or institution. About 21% had participated in at least one such mechanism. Compared to Sri Lanka, more respondents were satisfied with their experiences (ca 68%, no significant gender difference).

Accountability – Sri Lanka and Nepal

The main variable for accountability included in the analysis asked the question whether perpetrators should be held accountable. In Sri Lanka, the survey also asked “Should any of

the following things happen to [Sri Lanka Army soldiers]/[LTTE soldiers] who perpetrated violence in the 1983-2009 conflict?" and listed a set of options (both punishment, more positive options, and neutral). In the table below, variables were constructed only including the negative options (linearly in Models 1 and 3; dichotomously in Models 2 and 4), These options are: Punish them; Put them in jail; See them in trials; Want to see them dead; Have them compensate victims, and They should confess their crimes. As shown below, there are no gender differences, thus supporting the main results.

	(1)<br>Punishing army (OLS)	(2)<br>Punishing army binary (logit)	(3)<br>Punishing LTTE (OLS)	(4)<br>Punishing LTTE binary (logit)
female	-0.224	0.139	1.051	0.0931
	(3.748)	(0.151)	(3.930)	(0.143)
affected	-0.114	0.040	0.551	0.0553**
	(0.652)	(0.028)	(0.683)	(0.0258)
ptsd14	5.614	-0.001	6.649	-0.311*
	(4.183)	(0.170)	(4.386)	(0.160)
physhealth	-1.355	0.001	-2.252	-0.149*
	(2.096)	(0.085)	(2.198)	(0.0820)
education	-2.792	0.229	4.709	0.261
	(4.919)	(0.197)	(5.157)	(0.187)
famfinance	0.367	0.157	1.463	-0.0757
	(2.464)	(0.099)	(2.583)	(0.0936)
muslim	-15.190***	0.856***	-17.94***	-0.0683
	(5.068)	(0.185)	(5.314)	(0.189)
tamil	-3.243	3.011***	-9.432*	0.415**
	(5.286)	(0.256)	(5.542)	(0.205)
Constant	5.464	-1.354***	-1.552	1.095***
	(8.982)	(0.364)	(9.417)	(0.348)
Observations	1,008	1,008	997	997

Standard errors in parentheses; *** p<0.01, ** p<0.05, * p<0.1

In Nepal, respondents were asked the question "Should any of the following things happen to those who perpetrated violence in the 1996-2006 conflict?", i.e. not specified to specific conflict actors. Respondents could choose among a list of options (both punishment, more positive options, and neutral). In the table below, variables were constructed only including the negative options. These options are: Punish them; Put them in jail; See them in trials; Have them compensate victims, and They should confess their crimes.

	(1) Punishment	(2) Punishment binary
female	0.692***	0.163
	(0.119)	(0.148)
affected	0.050**	0.054
	(0.023)	(0.033)
ptsd14	-0.084	0.023
	(0.130)	(0.167)
physhealth	-0.325***	-0.177**
	(0.065)	(0.084)
education	-0.399***	0.036
	(0.118)	(0.148)
famfinance	-0.237***	-0.171
	(0.083)	(0.108)
highconflict	-0.336***	0.006
	(0.121)	(0.150)
/cut1	-2.767***	
	(0.301)	
/cut2	-1.273***	
	(0.291)	
/cut3	-0.741**	
	(0.288)	
/cut4	-0.391	
	(0.287)	
/cut5	0.266	
	(0.288)	
Constant		1.802***
		(0.389)
Observations	996	996

Standard errors in parentheses; *** $p<0.01$, ** $p<0.05$, * $p<0.1$

As reported in this specification, there is some support that women in Nepal are more likely to be positive about punishing perpetrators (as found in Model 1).

Peace accord views (Nepal)

The survey did not include any alternative questions about how people view the peace agreement in Nepal.

Additional control variables

Below, we report the results for all main regression models presented in the article, with the addition of three control variables. First, an important buffer in dealing with everyday insecurity and traumatic events is to have a social network. *Support from social network*: combines the two questions "How often do you have someone to confide in or talk to about yourself or your problems?" and "When I feel lonely, abandoned, sad, deeply upset, feel down,

angry, frustrated for different reasons I seek support from: " and is coded (1) if the respondent answers 'Some of the time; Most of the time; and All of the time' to the first question and selects at least one option for the second question). Second, we control for *Marital status*, coded (1) if married (0) if not (single, deserted by spouse, separated, divorced, widowed, spouse/partner missing). Third, a control for age is included to account for if the relationships are conditioned by being old enough to having lived through the armed conflict. *Age* is coded (1) if 31 or above (0) if 30 or below.

Below we report these results with regards to trust and coexistence, starting with Sri Lanka.

Trust and coexistence – Sri Lanka

	(1)<br>Trust<br>Sinhala	(2)<br>Trust<br>Tamil	(3)<br>Trust<br>Muslim	(4)<br>Threat<br>Sinhala	(5)<br>Threat<br>Tamil	(6)<br>Threat<br>Muslim	(7)<br>Comfortable<br>Army	(8)<br>Comfortable ex-<br>LTTE
female	-0.251	-0.567***	-0.557***	0.136	0.035	0.042	-0.663***	-0.465**
	(0.175)	(0.178)	(0.181)	(0.159)	(0.169)	(0.171)	(0.211)	(0.235)
affected	-0.043	0.040	0.003	0.113***	0.023	0.012	-0.077**	0.107**
	(0.027)	(0.028)	(0.027)	(0.025)	(0.028)	(0.026)	(0.037)	(0.041)
ptsd14	0.049	0.408**	0.453**	0.052	0.035	0.267	0.106	-0.071
	(0.182)	(0.192)	(0.207)	(0.166)	(0.184)	(0.194)	(0.236)	(0.263)
physhealth	-0.050	0.029	0.019	-0.173*	0.036	-0.055	-0.052	-0.001
	(0.101)	(0.100)	(0.096)	(0.094)	(0.096)	(0.092)	(0.119)	(0.133)
education	-0.302	0.118	0.141	0.265	-0.243	-0.007	0.044	0.634**
	(0.241)	(0.234)	(0.243)	(0.212)	(0.221)	(0.231)	(0.280)	(0.312)
famfinance	0.020	0.137	0.081	0.174*	0.097	0.040	0.059	0.143
	(0.117)	(0.113)	(0.117)	(0.106)	(0.108)	(0.111)	(0.138)	(0.154)
network	0.298	0.109	-0.437	0.155	-0.082	0.145	0.652*	-0.336
	(0.285)	(0.304)	(0.382)	(0.251)	(0.280)	(0.333)	(0.375)	(0.422)
married	0.226	0.270	0.338	-0.043	-0.009	0.045	-0.364	-0.064
	(0.252)	(0.286)	(0.256)	(0.228)	(0.267)	(0.241)	(0.312)	(0.350)
agebinary	0.551**	-0.118	0.061	-0.811***	-0.474**	-0.389*	0.410	0.241
	(0.240)	(0.230)	(0.246)	(0.213)	(0.220)	(0.232)	(0.288)	(0.323)
muslim							-5.314***	-0.356
							(0.290)	(0.324)
tamil							-6.278***	3.274***
							(0.300)	(0.336)
/cut1	-0.934	-0.272	-0.407	-0.234	-0.313	-0.237		
	(0.586)	(0.580)	(0.625)	(0.538)	(0.550)	(0.576)		
/cut2	2.042***	2.640***	2.924***	1.641***	1.987***	1.786***		
	(0.592)	(0.594)	(0.646)	(0.542)	(0.559)	(0.583)		
Constant							9.885***	1.941**
							(0.710)	(0.796)
Observations	540	544	515	581	559	524	969	967
R-squared							0.457	0.205

Standard errors in parentheses; *** p<0.01, ** p<0.05, * p<0.1

The results are in line with those reported in the article. Interestingly, younger respondents (30 or younger) report less perceived threats.

Next, we move on to Nepal.

Trust and coexistence – Nepal

	(1) Trust Maoists	(2) Trust Army	(3) Threat Maoists	(4) Threat Army	(5) Comfortable Maoists
female	-0.044	-0.339**	0.773***	0.778***	-1.069***
	(0.136)	(0.136)	(0.145)	(0.143)	(0.302)
affected	0.054**	-0.072***	0.011	0.075***	0.115*
	(0.027)	(0.027)	(0.029)	(0.028)	(0.060)
ptsd14	0.077	0.110	0.262*	0.191	-0.332
	(0.150)	(0.147)	(0.153)	(0.151)	(0.327)
physhealth	-0.035	-0.021	0.139*	0.029	0.012
	(0.074)	(0.075)	(0.081)	(0.078)	(0.165)
education	-0.024	0.363***	0.162	0.207	-0.696**
	(0.138)	(0.139)	(0.145)	(0.145)	(0.310)
famfinance	-0.022	0.143	0.080	-0.053	-0.104
	(0.095)	(0.094)	(0.099)	(0.099)	(0.206)
network	0.718*	0.914**	0.291	0.644	2.602***
	(0.384)	(0.371)	(0.396)	(0.424)	(0.758)
married	0.259	0.159	-0.473**	-0.470**	0.258
	(0.198)	(0.191)	(0.195)	(0.193)	(0.412)
agebinary	0.103	-0.106	-0.368**	-0.204	0.768**
	(0.157)	(0.156)	(0.159)	(0.157)	(0.349)
highconflict	-0.005	-0.129	0.430***	0.420***	-0.384
	(0.136)	(0.135)	(0.145)	(0.143)	(0.302)
/cut1	0.727	0.071	1.463***	1.316**	
	(0.520)	(0.512)	(0.557)	(0.559)	
/cut2	3.031***	2.530***	3.234***	3.314***	
	(0.530)	(0.519)	(0.566)	(0.569)	
Constant					7.099***
					(1.086)
Observations	888	905	902	916	951
R-squared					0.054

Standard errors in parentheses; *** p<0.01, ** p<0.05, * p<0.1

The results are in line with the main results.

In the next table, additional control variables are added to the analyses of truthtelling, in both cases.

Truthtelling – Sri Lanka and Nepal

	Sri Lanka		Nepal	
	(1) Collecting testimonies	(2) Sharing truth	(3) Collecting testimonies	(4) Sharing truth
female	0.174	-0.280**	-0.401***	-0.214
	(0.126)	(0.126)	(0.155)	(0.147)
affected	0.070***	0.011	-0.015	0.027
	(0.023)	(0.022)	(0.030)	(0.029)
ptsd14	-0.098	-0.025	0.190	0.181
	(0.142)	(0.139)	(0.171)	(0.158)
physhealth	0.060	0.069	-0.191**	-0.140*
	(0.071)	(0.072)	(0.088)	(0.081)
education	0.101	0.225	0.526***	0.060
	(0.166)	(0.165)	(0.158)	(0.151)
famfinance	-0.017	-0.039	-0.245**	-0.146
	(0.083)	(0.082)	(0.111)	(0.106)
network	0.273	0.315	0.570	0.325
	(0.219)	(0.223)	(0.399)	(0.385)
married	-0.236	-0.221	-0.243	-0.184
	(0.189)	(0.191)	(0.220)	(0.214)
agebinary	-0.065	0.057	0.216	-0.175
	(0.171)	(0.173)	(0.175)	(0.169)
muslim	-0.305*	0.037		
	(0.173)	(0.174)		
tamil	0.313*	-0.267		
	(0.178)	(0.177)		
highconflict			0.231	-0.205
			(0.155)	(0.149)
/cut1	-1.166***	-1.786***	-3.084***	-3.763***
	(0.425)	(0.427)	(0.595)	(0.570)
/cut2	-0.671	-1.269***	-2.587***	-3.179***
	(0.422)	(0.423)	(0.591)	(0.562)
/cut3	0.714*	0.438	-1.325**	-1.380**
	(0.423)	(0.421)	(0.584)	(0.551)
Observations	929	936	891	873

Standard errors in parentheses; *** p<0.01, ** p<0.05, * p<0.1

These results again substantially support those reported in the article, for both countries. Next, we move on to the analysis of accountability of perpetrators, with the additional control variables added.

Accountability – Sri Lanka and Nepal

	(1)<br>Sri Lanka	(2)<br>Nepal
female	-0.005	-0.107
	(0.139)	(0.155)
affected	0.049*	-0.022
	(0.025)	(0.030)
ptsd14	-0.117	0.210
	(0.155)	(0.169)
physhealth	-0.017	-0.178**
	(0.079)	(0.087)
education	0.477***	-0.022
	(0.179)	(0.160)
famfinance	0.092	-0.109
	(0.091)	(0.110)
network	-0.040	-1.245**
	(0.257)	(0.624)
married	0.205	0.326
	(0.207)	(0.212)
agebinary	-0.212	0.306*
	(0.193)	(0.175)
muslim	0.667***	
	(0.191)	
tamil	0.836***	
	(0.197)	
highconflict		-0.224
		(0.159)
/cut1	-1.232***	-4.238***
	(0.475)	(0.754)
/cut2	-0.679	-3.706***
	(0.472)	(0.750)
/cut3	0.581	-2.542***
	(0.471)	(0.745)
Observations	923	854

Standard errors in parentheses;
*** p<0.01, ** p<0.05, * p<0.1

Again, these results are substantially similar to those reported in the article, for both cases. Last, we report the results regarding views on the Comprehensive Peace Agreement in Nepal with the inclusion of additional control variables.

Peace accord views (Nepal)

	(1) CPA necessary	(2) CPA reflects Nepalis
female	0.131	-0.065
	(0.363)	(0.244)
affected	-0.073	-0.007
	(0.058)	(0.042)
ptsd14	0.173	-0.155
	(0.365)	(0.243)
physhealth	-0.321	-0.203
	(0.200)	(0.132)
education	0.662*	-0.117
	(0.361)	(0.258)
famfinance	-0.157	-0.298*
	(0.243)	(0.167)
network	0.019	-0.303
	(1.115)	(0.838)
married	0.968**	0.143
	(0.454)	(0.344)
agebinary	0.392	0.263
	(0.403)	(0.265)
highconflict	0.039	0.087
	(0.358)	(0.238)
/cut1	-4.104***	-5.179***
	(1.537)	(1.129)
/cut2	-3.548**	-4.411***
	(1.519)	(1.109)
/cut3	-2.058	-2.383**
	(1.499)	(1.089)
Observations	419	411

Standard errors in parentheses; *** p<0.01, ** p<0.05, * p<0.1

Also in these regressions, the results hold as in the main models.

Trimmed models

Many of the control variables are not significant in most models. Below are the main tables from the article, but with only the theoretically relevant IV (gender), and the control variables that showed significance along with variables included for spatial dependence.

Trust and coexistence – Sri Lanka

	(1) Trust Sinhala	(2) Trust Tamil	(3) Trust Muslim	(4) Threat Sinhala	(5) Threat Tamil	(6) Threat Muslim	(7) Comfortable Army	(8) Comfortable ex-LTTE
female	-0.190	-0.595***	-0.537***	0.162	0.028	0.102	-0.718***	-0.494**
	(0.170)	(0.171)	(0.177)	(0.155)	(0.160)	(0.163)	(0.205)	(0.229)
affected				0.102***			-0.065*	0.082**
				(0.024)			(0.034)	(0.039)
education	-0.468**			0.390*				
	(0.220)			(0.200)				
famfinance				0.199**				
				(0.101)				
ptsd14		0.407**	0.426**					
		(0.177)	(0.182)					
muslim							-5.435***	-0.336
							(0.272)	(0.304)
tamil							-6.331***	3.372***
							(0.282)	(0.316)
/cut1	-1.546***	-1.183***	-0.670***	0.848**	-0.114	-0.169		
	(0.225)	(0.143)	(0.138)	(0.331)	(0.117)	(0.120)		
/cut2	1.369***	1.751***	2.621***	2.682***	2.168***	1.871***		
	(0.222)	(0.157)	(0.203)	(0.349)	(0.159)	(0.151)		
Constant							10.546***	2.617***
							(0.220)	(0.245)
Obs	868	894	845	917	900	850	992	990
R-squared							0.458	0.203

Standard errors in parentheses; *** p<0.01, ** p<0.05, * p<0.1

The results are substantially the same as in the main models. Next, we examine trimmed models regarding trust and coexistence with regards to Nepal.

Trust and coexistence – Nepal

	(1)<br>Trust Maoists	(2)<br>Trust Army	(3)<br>Threat Maoists	(4)<br>Threat Army	(5)<br>Comfortable Maoists
female	-0.054	-0.310**	0.757***	0.784***	-1.044***
	(0.129)	(0.128)	(0.136)	(0.135)	(0.289)
affected	0.068***	-0.069***	0.003	0.078***	0.143**
	(0.025)	(0.025)	(0.027)	(0.026)	(0.057)
education	-0.048	0.425***	0.425***	0.377***	-1.032***
	(0.128)	(0.128)	(0.134)	(0.133)	(0.288)
highconflict	-0.054	-0.146	0.454***	0.424***	-0.480
	(0.133)	(0.131)	(0.140)	(0.138)	(0.297)
/cut1	-0.138	-1.162***	1.241***	1.316***	
	(0.150)	(0.155)	(0.161)	(0.162)	
/cut2	2.129***	1.269***	2.965***	3.264***	
	(0.172)	(0.156)	(0.187)	(0.195)	
Constant					10.078***
					(0.326)
Observations	907	926	923	938	981
R-squared					0.033

Standard errors in parentheses; *** p<0.01, ** p<0.05, * p<0.1

The results are substantially the same as in the main models. Next, we move on to truthtelling.

Truthtelling – Sri Lanka and Nepal

	Sri Lanka		Nepal	
	(1)<br>Collecting testimonies	(2)<br>Sharing truth	(3)<br>Collecting testimonies	(4)<br>Sharing truth
female	0.079	-0.262**	-0.442***	-0.186
	(0.121)	(0.121)	(0.148)	(0.140)
physhealth			-0.221***	-0.143*
			(0.085)	(0.079)
education			0.498***	0.153
			(0.149)	(0.142)
famfinance			-0.263**	-0.161
			(0.109)	(0.103)
highconflict			0.220	-0.154
			(0.148)	(0.143)
muslim	-0.116	0.045		
	(0.147)	(0.150)		
tamil	0.518***	-0.218		
	(0.148)	(0.146)		
/cut1	-1.576***	-2.227***	-3.756***	-3.870***

	(0.135)	(0.149)	(0.393)	(0.378)
/cut2	-1.077***	-1.713***	-3.272***	-3.288***
	(0.126)	(0.137)	(0.386)	(0.366)
/cut3	0.296**	-0.006	-2.019***	-1.500***
	(0.121)	(0.123)	(0.373)	(0.349)
Observations	957	965	902	882

Results are similar to those reported in the article. In the following table, we examine accountability.

Accountability – Sri Lanka and Nepal

	(1)<br>Sri Lanka	(2)<br>Nepal
female	-0.067	-0.160
	(0.135)	(0.146)
affected	0.045*	
	(0.024)	
physhealth		-0.262***
		(0.078)
education	0.498***	
	(0.161)	
muslim	0.603***	
	(0.179)	
tamil	0.751***	
	(0.186)	
highconflict		-0.228
		(0.150)
/cut1	-1.394***	-3.470***
	(0.206)	(0.315)
/cut2	-0.832***	-2.944***
	(0.197)	(0.306)
/cut3	0.451**	-1.786***
	(0.194)	(0.294)
Observations	945	868

Standard errors in parentheses
*** p<0.01, ** p<0.05, * p<0.1

Again, results are similar to those reported in the article. Last, we examine views regarding the peace agreement in Nepal.

Peace accord views (Nepal)

	(1) CPA necessary	(2) CPA reflects Nepalis
female	-0.015	-0.111
	(0.330)	(0.228)
physhealth	-0.380*	-0.234*
	(0.195)	(0.127)
famfinance	-0.092	-0.262
	(0.237)	(0.162)
highconflict	-0.105	0.117
	(0.335)	(0.228)
/cut1	-5.655***	-5.023***
	(0.945)	(0.637)
/cut2	-5.105***	-4.254***
	(0.913)	(0.599)
/cut3	-3.595***	-2.204***
	(0.875)	(0.562)
Observations	423	415

Standard errors in parentheses; *** p<0.01, ** p<0.05, * p<0.1

Again, the results from the main models remain in these trimmed models.
